# Supplementary figures and images for: Trim28 Haploinsufficiency Triggers Bi-stable Epigenetic Obesity
Source: Cell. 2016 Jan 28;164(3):353–64. doi: 10.1016/j.cell.2015.12.025 (PMC4735019; doi:10.1016/j.cell.2015.12.025)

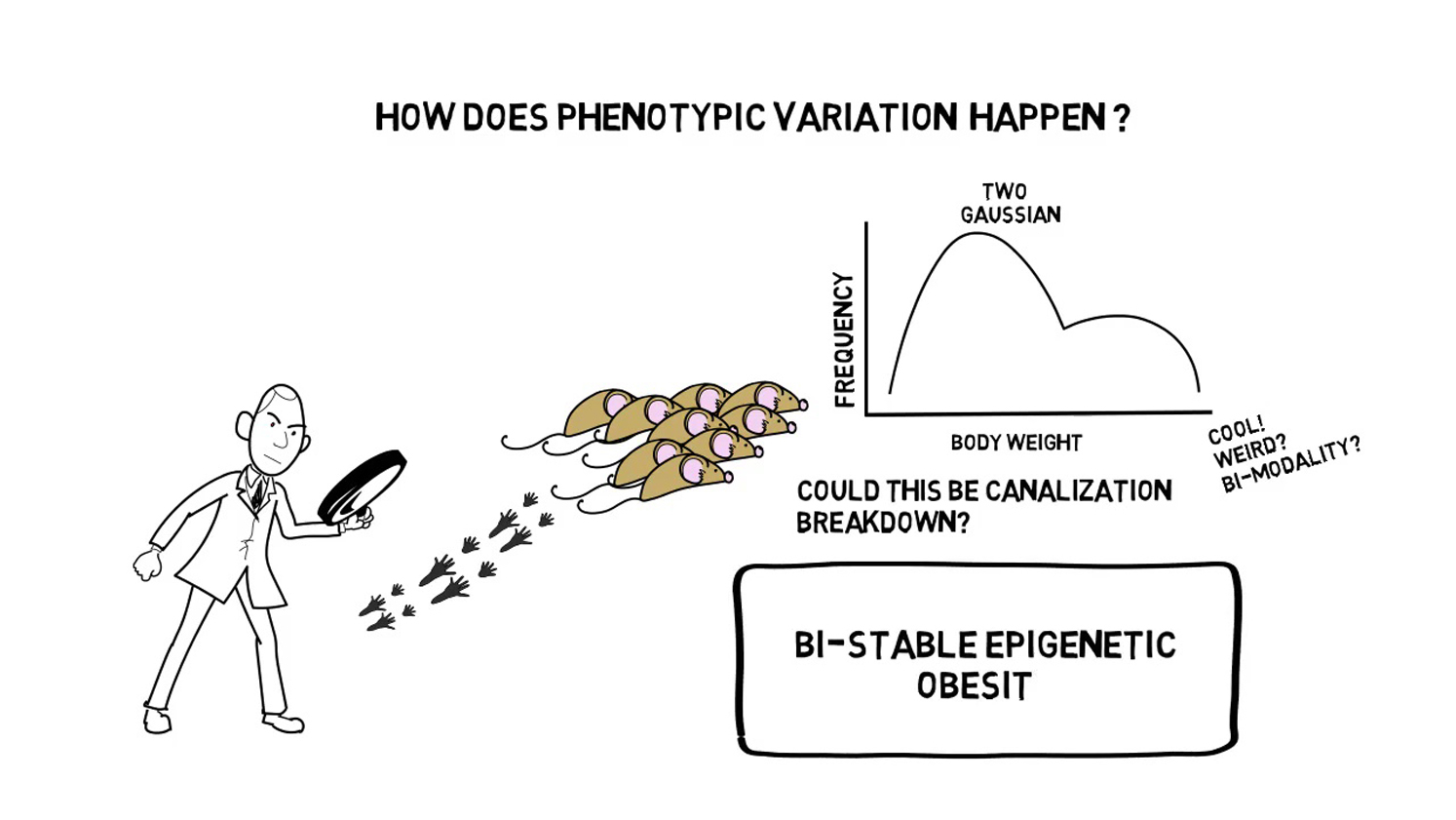

Supplement: Supplementary file 1 [file mmc8.jpg]
