# Supplementary material for: Trim28 Haploinsufficiency Triggers Bi-stable Epigenetic Obesity
Source: Cell. 2016 Jan 28;164(3):353–64. doi: 10.1016/j.cell.2015.12.025 (PMC4735019; doi:10.1016/j.cell.2015.12.025)
Supplement: Document S1. Supplemental Experimental Procedures [file mmc1.pdf]

Cell

Supplemental Information

Trim28 Haploinsufficiency Triggers Bi-stable Epigenetic Obesity

Kevin Dalgaard, Kathrin Landgraf, Steffen Heyne, Adelheid Lempradl, John Longinotto, Klaus Gossens, Marius Ruf, Michael Orthofer, Ruslan Strogantsev, Madhan Selvaraj, Tess Tsai-Hsiu Lu, Eduard Casas, Raffaele Teperino, M. Azim Surani, Ilona Zvetkova, Debra Rimmington, YC Loraine Tung, Brian Lam, Rachel Larder, Giles SH Yeo, Stephen O'Rahilly, Tanya Vavouri, Emma Whitelaw, Josef M. Penninger, Thomas Jenuwein, Ching-Lung Cheung, Anne C. Ferguson-Smith, Anthony P. Coll, Antje Körner, and J. Andrew Pospisilik

SUPPLEMENTAL EXPERIMENTAL PROCEDURES

Animal Husbandry

All mice were maintained under controlled temperature (22°C) and on a 12hr light, 12hr dark schedule (light on 6:00-18:00). Food and water were available *ad libitum* unless otherwise stated. All mice were weaned at 3 weeks of age onto a standard chow (SDS RM3, Essex, UK or V1185-300 MZ-Ereich, ssniff, Germany). Body composition was determined using dual-energy X-ray absorptiometry (DEXA, PIX-Imus2 Series Densimeters) under terminal anaesthetic (Dolethal, Vetoquinol UK Ltd). All protocols were in accordance with German and United Kingdom legislation; Project license numbers 80/2098, 80/2497, and 35-9185.81/G-10/94.

Generation of *Trim28* and *Nnat* Heterozygous Mice

The generation of Trim28$^{+/D9}$ is described elsewhere (Blewitt et al, 2005 and Whitelaw et al, 2010). Briefly, the line was generated by ENU mutation in an FVB/NJ congenic line expressing a GFP transgene array (Whitelaw et al 2010). This mouse line was outcrossed 2 times to C57BL6/J for mutation mapping, then, backcrossed to FVB/NJ for more than 10 generations and inbred another 20+ generations. The Nnat targeting vector was designed to produce a “KO first allele” (reporter-tagged insertion with conditional potential) (Figure 3A). A Gateway adapted intermediate vector containing Nnat homology arms and the critical exons 2 and 3 was obtained from The Wellcome Trust Sanger Institute high throughput gene targeting pipeline, design ID 44045 (http://www.sanger.ac.uk/htgt/htgt2/design/designlist/list_designs). A Gateway reaction was assembled with Nnat-interm_44045 vector, pL3L4_DONR223_Spec_DTA(-) Terminator and pL1L2_GTIRES_BetactP_FLFL provided by Dr B. Rosen (The Wellcome Trust Sanger Institute). The resulting vector was used to target JM8.F6 C57Bl6/N embryonic stem cells cultured according to standard protocols as recommended by the International Knockout Mouse Consortium. Confirmation of targeting was by PCR with primer sets 21_16 and 9_19 (available on request). Targeting efficiency was 40%.

Proper targeting was confirmed for clones A6 and B12 by Southern blot and clones were injected into to C57BL/6J-Tyr^{c-2J} (albino) blastocysts. Chimeric mice were then mated with C57BL/6J-Tyr^{c-2J} mice and germ line transmission was validated by the appearance of black offspring. The founder mice were then crossed with C57BL/6J mice. Targeted mice were genotyped by PCR using primer sets 55_56_57 and 59_60_61 (available on request). Mice heterozygous for the “KO first allele” were crossed with CMVCre C57BL/6J mice (kind gift from Dr M. Constancia) to delete the *neo* cassette and exons 2 and 3 from the targeted *Nnat* genomic locus. Primer set 82_83 was used to

confirm recombination between loxP site 1 and loxP site 3. Mice carrying the *Nnat* KO allele were crossed with C57BL/6J mice to breed the *Cre* transgene out. qPCR and Western blotting were used to confirm the lack of *Nnat* expression in the targeted mice (Figures 3B and 3C). $Nnat^{+/-}$ mice were backcrossed for 10 generations onto a C57BL/6J background before undergoing phenotypic analysis. $Nnat^{+/-p}$ animals were generated by crossing wild-type females with male $Nnat^{+/-}$ mice. $Nnat^{+/-m}$ mice were generated by crossing $Nnat^{+/-}$ females with wild-type male mice. $Nnat^{+/-m}$ mice were phenotypically identical to $Nnat^{+/+}$ mice (Figure 3D).

Generation of *Trim28* Tissue-specific Knockout Mice

The generation of animals harboring conditional *Trim28* alleles has been previously described (Cammas et al, 2000). Constitutive gene inactivation in tissues was performed using transgenic Alb-Cre (liver), McK-Cre (muscle), adipose (Adipoq-Cre), POMC (POMC-Cre) and AgRP (AgRP-Cre) animals obtained from The Jackson Laboratory. To generate tissue-specific knockouts, $Trim28^{flox/flox}$ animals were crossed with Cre-positive $Trim28^{+/flox}$ animals to obtain mice homozygous for the floxed *Trim28* locus. These crosses produce Cre-positive-control and tissue-specific heterozygote and knockout littermates. Data shown are from 14-18 weeks old animals.

Quantitative PCR

Total RNA was extracted using TRI Reagent (SIGMA) and was reverse-transcribed into cDNA using commercially available kits (Applied Biosystems). qPCR reactions were performed a 7900HT Fast Real-Time PCR System (Applied Biosystems). Post-amplification melting curve analysis was performed to check for unspecific products and primer-only controls were included to ensure the absence of primer dimers. For normalization threshold cycles (Ct-values) were normalized to either TBP (mouse samples) or to ACTB, HPRT and TBP (human samples) within each sample to obtain sample-specific ΔCt values (= Ct *gene of interest* - Ct *housekeeping gene*). $2^{-\Delta\Delta Ct}$ values were calculated to obtain fold expression levels, where ΔΔCt = (ΔCt treatment - ΔCt control). Human transcripts were quantified using TaqMan gene expression assays with validated probes (Life Technologies). Human and mouse primers used are listed in Supplemental Table S5.

Glucose Tolerance Test

Following an overnight fast, mice were administered glucose (1 g/kg) by oral gavage, and blood samples for glucose measurement were collected from the tail vein at the indicated times. Glycemia was assessed using a OneTouch Vita glucometer.

Mouse Laboratory Parameters and Cytokines

Free fatty acids and glycerol were measured using the non-esterified fatty acid (NEFA) kit (Wako Chemicals) and Triglyceride Determination kit (SIGMA), respectively, and the provided protocol was followed. Serum leptin, TNFα, CRF, RAGE and Resistin levels were quantified using commercially available ELISA kits (Millipore, R&D Systems).

Histology, Adipocyte Size and Number

For tissue sections, hematoxylin and eosin (H&E) staining was performed on 2 µm paraffin sections of tissues fixed in 4% phosphate-buffered formalin. Adipocyte size distribution was determined by semi-automated morphometry. In brief, whole fat pads were visualized using whole slide scans captured by the Mirax Scan Pannoramic Scanner with a 20x objective (Zeiss and 3DHistec). Four fields of view of 200 µm intervals per animal were quantified. Epididymal fat pads from 4 animals per group were analyzed. Semi-automated morphometry (ImageJ, plugin Adipocytes Tool) was used to define and quantify adipocytes based on shape, size and presence of a lipid droplet.

Immunofluorescence

Paraffinized sections were heated, deparaffinized and rinsed in water. Antigen retrieval was performed by heating the slides at 95°C for 20 minutes in HistoVT pH 7.0 (Nacalai USA). Specimens were blocked in 5% goat serum PBS-T and incubated with Nnat primary antibody (1:50 dilution) (AB27266, Abcam) for 48 hours. Specimens were incubated in fluorochrome-conjugated secondary antibody. VectorShield with DAPI and coverslip were mounted and slides were allowed to cure overnight at 4°C in the dark before image acquisition. Images were acquired using LSM780 (Zeiss).

RNA Sequencing

Trizol-purified RNA was poly(A)-enriched and libraries prepared using the TruSeq Sample Prep v2 kit (Illumina) and sequenced on an Illumina HiSeq 2500 sequencer. All mouse sequence data were performed in at least three biological replicates at 2 x 50bp length with high quality metrics (> 20 Phred score) and nucleotide distribution. > 15 million reads per mouse sample and 10 million reads per human sample were mapped using TopHat v2.0.8b with -G option against *Mus musculus* genome (mm9, iGenome UCSC) and *Homo sapiens* genome (hg19, iGenome UCSC), respectively. Gene expression values and significantly differentially expressed genes were calculated using Cuffnorm and Cuffdiff v2.2.1 (Trapnell et al., 2013) with geometric normalization and multi-read correction (-u option).

Reduced Representative Bisulfite Sequencing

Total genomic DNA was digested over night with MspI (NEB) and sequencing libraries were prepared with NEBNext DNA Library kit (NEB). Libraries were size-selected (150-350 bp) on an agarose gel followed by bisulfite conversion using Imprint DNA Modification kit (SIGMA) using the 2-step protocol (2 hr at 65°C). Samples were PCR amplified using KAPA Hifi Uracil+ high-fidelity polymerase (KAPA Biosystems). Library quality was assessed using KAPA Library Quantification kit (KAPA BIosystem) and sequenced on an Illumina HiSeq 2500 sequencer. Data were analyzed using RnBeads V.0.99.16 (Assenov et al., 2014).

Quantitative Bisulfite Pyrosequencing

Total genomic DNA was bisulfite converted using Imprint DNA Modification kit (SIGMA) using the 2-step protocol (2 hr at 65°C). Samples were PCR amplified for the region of interest and bound to streptavidin beads using PyroMark Binding Buffer (Qiagen). Beads were washed in 70% Ethanol, 0.4N NaOH and 10mM Tris-acetate (pH 7.6) using a PyroMark Vacuum workstation (Qiagen). DNA-bound beads were resuspended in PyroMark annealing buffer (Qiagen) containing 10uM Pyrosequencing primer (PSQ, Qiagen). Samples were heated up and sequenced on a PyroMark Q96 PyroSequencer (Qiagen) in triplicates and were analysed using PyroMark Q96 ID Software 2.5.

Human Study Population

Study subjects of the Leipzig Childhood adipose cohort (NCT02208141) included 18 obese patients and 22 lean controls aged 2-18 years that underwent elective orthopedic surgery, herniotomy/orchidopexie or other surgeries (Landgraf et al., 2015). Participants were included if they were free of severe diseases and medication potentially affecting adipose tissue biology. The following exclusion criteria were applied: diabetes, generalized inflammation, malignant diseases, genetic syndromes, or permament immobilization. Written informed consent was obtained from all parents and study protocols were approved by the local Ethics Committee (265–08, 265–08-ff). BMI data of study participants were standardized to age- and sex-specific centiles applying German reference data (Kromeyer-Hauschild et al., 2001). Overweight and obesity are defined by a cutoff of 1.28 and 1.88 SDS (90th or 97th centile), respectively. Three overweight and 15 obese subjects were combined to form the “Obese” group. Tissue biopsies from subcutaneous adipose tissue were excised during surgery, washed three times in PBS, and immediately frozen in liquid nitrogen for RNA isolation.

Reagents

Unless otherwise stated all chemicals and reagents were obtained from SIGMA.

Statistical Analysis

Data are expressed as mean ± standard error of the mean (SEM) unless otherwise specified. Statistical significance was tested by Student’s t-test or ANOVA where appropriate. Correlations were tested by linear regression. All figures and mouse statistical analyses were generated using Prism 5 (GraphPad). All reported p-values are two-tailed unless stated otherwise. $p < 0.05$ was considered to indicate statistical significance.

Supplemental References

Assenov, Y., Müller, F., Lutsik, P., Walter, J., Lengauer, T., and Bock, C. Comprehensive analysis of DNA methylation data with RnBeads. *Nat Methods* 11, 1138-140 (2014).

Cammas F, Mark M, Dollé P, Dierich A, Chambon P, Losson R. Mice lacking the transcriptional corepressor TIF1beta are defective in early postimplantation development. *Development. 2000 Jul;127(13):2955-63.*

Kromeyer-Hauschild, K., Wabitsch, M., Kunze, D., Geller, F., Geiß, H.C., Hesse, V., von Hippel, A., Jaeger, U., Johnsen, D., and Korte, W. Perzentile für den Body-mass-Index für das Kindes-und Jugendalter unter Heranziehung verschiedener deutscher Stichproben. *Monatsschrift Kinderheilkunde* 149, 807-818 (2001).

Landgraf, K., Rockstroh, D., Wagner, I.V., Weise, S., Tauscher, R., Schwartze, J.T., Löffler, D., Bühligen, U., Wojan, M., Till, H., Kratzsch, J., Kiess, W., Blüher, M., and Körner, A. Evidence of early alterations in adipose tissue biology and function and its association with obesity-related inflammation and insulin resistance in children. *Diabetes* 64, 1249-261 (2015).

Trapnell, C., Hendrickson, D.G., Sauvageau, M., Goff, L., Rinn, J.L., and Pachter, L. Differential analysis of gene regulation at transcript resolution with RNA-seq. *Nat Biotechnol* 31, 46-53 (2013).
