# Supplementary material for: Trim28 Haploinsufficiency Triggers Bi-stable Epigenetic Obesity
Source: Cell. 2016 Jan 28;164(3):353–64. doi: 10.1016/j.cell.2015.12.025 (PMC4735019; doi:10.1016/j.cell.2015.12.025)
Supplement: Document S2. Article plus Supplemental Information [file mmc7.pdf]

Cell

Article

Trim28 Haploinsufficiency Triggers Bi-stable Epigenetic Obesity

Graphical Abstract

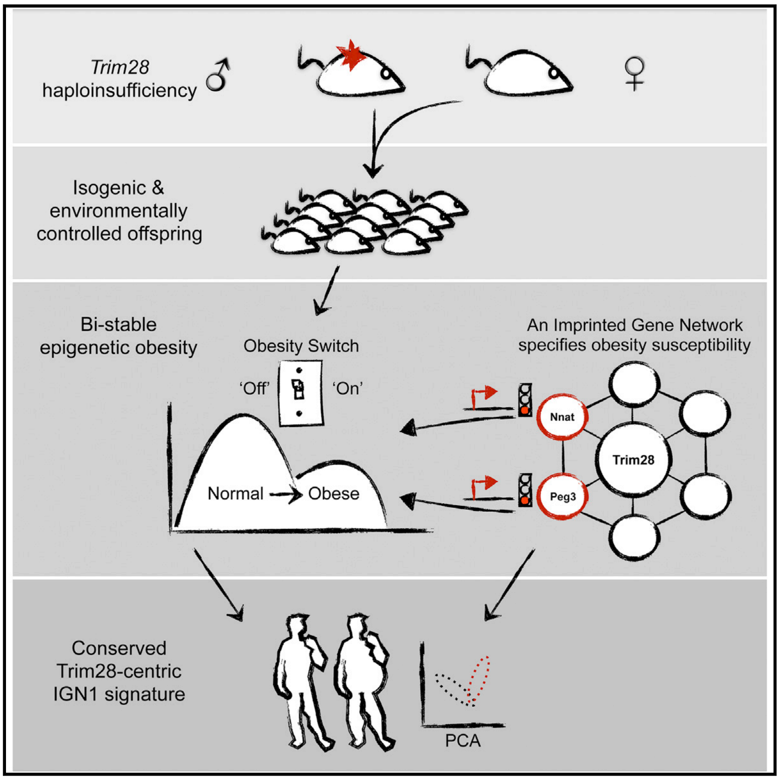

Highlights

- *Trim28* haploinsufficiency triggers stochastic bi-stable obesity or polyphenism
- Non-classical imprinted gene dysregulation specifies "on" versus "off" obese states
- *Peg3* and *Nnat* perturbation trigger stochastic bi-stable obesity
- Human BMI distributions and transcriptomes suggest Trim28-associated subpopulations

Authors

Kevin Dalgaard, Kathrin Landgraf, Steffen Heyne, ..., Anthony P. Coll, Antje Körner, J. Andrew Pospisilik

Correspondence

pospisilik@ie-freiburg.mpg.de

In Brief

TRIM28 insufficiency in both mouse and human leads to polyphenism, wherein lean and obese phenotypes can arise from the identical genotypes through dysregulation of an imprinted gene network.

Dalgaard et al., 2016, Cell *164*, 353–364
January 28, 2016 ©2016 The Authors
http://dx.doi.org/10.1016/j.cell.2015.12.025

Cell

Trim28 Haploinsufficiency Triggers Bi-stable Epigenetic Obesity

Kevin Dalgaard,[1,13] Kathrin Landgraf,[2,3] Steffen Heyne,[1] Adelheid Lempradl,[1] John Longinotto,[1] Klaus Gossens,[1] Marius Ruf,[1] Michael Orthofer,[4] Ruslan Strogantsev,[5] Madhan Selvaraj,[1] Tess Tsai-Hsiu Lu,[1] Eduard Casas,[6] Raffaele Teperino,[1,14] M. Azim Surani,[7,8,9] Ilona Zvetkova,[10] Debra Rimmington,[10] Y.C. Loraine Tung,[10] Brian Lam,[10] Rachel Larder,[10] Giles S.H. Yeo,[10] Stephen O'Rahilly,[10] Tanya Vavouri,[6] Emma Whitelaw,[11] Josef M. Penninger,[4] Thomas Jenuwein,[1] Ching-Lung Cheung,[12] Anne C. Ferguson-Smith,[5] Anthony P. Coll,[10] Antje Körner,[2,3] and J. Andrew Pospisilik[1,*]

[1]Max Planck Institute of Immunobiology and Epigenetics, Stübeweg 51, 79108 Freiburg, Germany
[2]Department of Women's and Child Health, Center for Pediatric Research Leipzig, University Hospital for Children & Adolescents
[3]Integrated Research and Treatment Center (IFB) Adiposity Diseases
University of Leipzig, 04103 Leipzig, Germany
[4]IMBA, Institute of Molecular Biotechnology of the Austrian Academy of Sciences,1030 Vienna, Austria
[5]Department of Genetics, University of Cambridge, Cambridge CB2 3EH, UK
[6]Institute for Predictive and Personalized Medicine of Cancer (IMPPC) and Josep Carreras Leukaemia Research Institute, Can Ruti Campus, Ctra de Can Rutí, Cami de les Escoles s/n, Badalona 08916, Barcelona, Spain
[7]Wellcome Trust Cancer Research UK Gurdon Institute, University of Cambridge, Tennis Court Road, Cambridge CB2 1QN, UK
[8]Department of Physiology, Development and Neuroscience, University of Cambridge, Downing Street, Cambridge CB2 3EG, UK
[9]Wellcome Trust-MRC Stem Cell Institute, University of Cambridge, Tennis Court Road, Cambridge CB2 3EG, UK
[10]University of Cambridge Metabolic Research Laboratories and MRC Metabolic Diseases Unit, Welcome Trust-MRC Institute of Metabolic Science, Addenbrooke's Hospital, Cambridge CB2 0QQ, UK
[11]Department of Genetics, La Trobe Institute for Molecular Science, La Trobe University, Bundoora, Melbourne, VIC 3086, Australia
[12]Department of Pharmacology and Pharmacy, Centre for Genomic Sciences, The University of Hong Kong, Hong Kong
[13]Present address: Novo Nordisk A/S, Novo Nordisk Park, 2760 Måløv, Denmark
[14]Present address: Institute of Experimental Genetics, Helmholtz Zentrum Muenchen and German Center for Diabetes Research (DZD), 85764 Neuherberg, Germany
*Correspondence: pospisilik@ie-freiburg.mpg.de
http://dx.doi.org/10.1016/j.cell.2015.12.025
This is an open access article under the CC BY license (http://creativecommons.org/licenses/by/4.0/).

SUMMARY

More than one-half billion people are obese, and despite progress in genetic research, much of the heritability of obesity remains enigmatic. Here, we identify a Trim28-dependent network capable of triggering obesity in a non-Mendelian, "on/off" manner. Trim28$^{+/D9}$ mutant mice exhibit a bi-modal body-weight distribution, with isogenic animals randomly emerging as either normal or obese and few intermediates. We find that the obese-"on" state is characterized by reduced expression of an imprinted gene network including *Nnat*, *Peg3*, *Cdkn1c*, and *Plagl1* and that independent targeting of these alleles recapitulates the stochastic bi-stable disease phenotype. Adipose tissue transcriptome analyses in children indicate that humans too cluster into distinct sub-populations, stratifying according to *Trim28* expression, transcriptome organization, and obesity-associated imprinted gene dysregulation. These data provide evidence of discrete *polyphenism* in mouse and man and thus carry important implications for complex trait genetics, evolution, and medicine.

INTRODUCTION

Complex traits such as height, shape, and weight emerge from the integration of multiple genetic and epigenetic determinants. They underpin susceptibility to and severity of virtually all disease.

Current estimates place obesity incidence at more than 600 million individuals worldwide (WHO, 2015). As a prime risk factor for heart disease, stroke, cancer, type 2 diabetes, and neurodegeneration, obesity poses a major socio-economic challenge. Although studies over the last decades have provided a genetic framework for understanding obesity, the contribution of epigenetic regulation remains poorly understood. Measurements in monozygotic twins and inbred mouse strains indicate that epigenetic control can have substantial effects on body-mass outcomes. Isogenic C57Bl6/J mice, for instance, can vary by as much as 100% in body weight when fed a high-fat diet, even when reared in highly standardized laboratory conditions (Koza et al., 2006). Experiments in multiple model organisms suggest that pre-conceptual and early-life environment contribute to variability by reproducibly shifting offspring phenotype (reviewed in Patti, 2013; Daxinger and Whitelaw, 2012; Rando and Simmons, 2015). Also, epidemiological data suggest that similar regulatory mechanisms determine human phenotypic outcomes. Despite many investigations, we still know little about the mechanisms by which developmental trajectories are canalized and how these states are reproducibly altered. Changes in imprinting

(Morgan et al., 1999), DNA methylation (Wolff et al., 1998; Waterland et al., 2006; Carone et al., 2010; Anway et al., 2005; Radford et al., 2014), and non-coding RNA expression (Rechavi et al., 2014; Rassoulzadegan et al., 2006; Gapp et al., 2014; Kiani et al., 2013; Seong et al., 2011; Shirayama et al., 2012; Lee et al., 2012; Greer et al., 2011; Ashe et al., 2012) have been implicated in altering phenotypic outcomes in model organisms, and there is evidence that chromatin states, coding RNAs, and chromatin-associated molecular machinery are important (Öst et al., 2014; Greer et al., 2011).

Chromatin provides the cell with a template for regulating genome output. Genetic screens have identified numerous families of proteins that generate and define chromatin composition (Hollick and Chandler, 2001; Schotta et al., 2003; Allshire et al., 1994), and more recent (epi)genomic efforts have revealed insights into how the genome is physically and functionally partitioned (Naumova and Dekker, 2010). In these contexts, the Whitelaw group used ENU mutagenesis to uncover novel chromatin regulators in the mouse (Daxinger et al., 2013). Among 42 "Momme" (*mo*difiers of *m*urine *me*tastable epialleles) mutants, the group identified and mapped MommeD9 (Trim28$^{+/D9}$) to a non-sense mutation in the chromatin-interacting protein *Trim28* (also known as *Tif1β* or *Kap1*). Trim28$^{+/D9}$ mutant mice stood out in that they exhibited exaggerated phenotypic variation specifically in body mass and adiposity (Whitelaw et al., 2010). TRIM28 is a large multi-domain protein that supports heterochromatin deposition and silencing by bridging interactions between KRAB-zinc finger transcription factors and histone de-acetylases (HDAC1/2) and methyltransferases (SETDB1). Homozygous *Trim28* deletion strains are early embryonic lethal, demonstrating critical requirements for the protein in development. Also, consistent with the exacerbated phenotypic variation reported for Trim28$^{+/D9}$ animals, conditional maternal deletion mutants of *Trim28* exhibit highly variable developmental abnormalities (Messerschmidt et al., 2012).

Here, we report the characterization of body-mass hyper-variability in Trim28$^{+/D9}$ mice. Interestingly, we find that isogenic Trim28$^{+/D9}$ mutant animals exhibit obesity in an "on/off" manner, emerging into adulthood as either obese or normal, and thus yielding a bi-modal body-weight distribution for the population. Individuals of the obese sub-population exhibit reduced expression of an imprinted gene network that includes *Nnat*, *Peg3*, *Cdkn1c*, and *Plagl1*, and we find that deletion of either *Nnat* or *Peg3* alone is sufficient to recapitulate the bi-modal adiposity phenotype. Examining transcriptional profiles of multiple cohorts, we find evidence that humans too segregate into two apparent sub-populations stratified by *Trim28* expression, high-dimensional transcriptome arrangement, and body-mass index. The data provide genetic evidence that mechanisms exist in mammals to canalize developmental and phenotypic outcome along discrete trajectories, a concept also known as *polyphenism*.

RESULTS

Trim28 Haploinsufficiency Induces a Stochastic Bi-stable Obesity

Whitelaw et al. previously reported exaggerated variability in body mass in Trim28$^{+/D9}$ haploinsufficient mice (Whitelaw et al., 2010). In an effort to understand the nature of the variable phenotype, we generated large cohorts of Trim28$^{+/D9}$ animals well-controlled for parental and offspring litter size (7–10 pups), lactation sufficiency, and housing conditions, factors all known to influence growth and metabolism and thus to add noise to phenotypic data. As examples, individually housed mice eat ~25% more than those housed in groups of 4; also, small litter size (calorie excess during lactation) is used as a model for metabolic reprogramming toward obesity.

Rather than simple amplification of random (Gaussian-distributed) variation, we found that highly backcrossed and inbred (F10+N20+ FVB/NJ) Trim28$^{+/D9}$ individuals clustered into two discrete sub-populations, one heavy (obese-Trim28$^{+/D9}$) and one normal (lean-Trim28$^{+/D9}$) (Figure 1A). The resulting body-weight frequency distribution was bi-modal (Figure 1B). Interestingly, this on/off phenotype was observed not only when merging data from multiple litters but also within individual litters, i.e., when parental and developmental environment are maximally controlled. In addition to increased body mass, the obese-Trim28$^{+/D9}$ sub-population was slightly longer (~1%–2% increase in nose-to-tail length; Figure 1C) relative to both their lean-Trim28$^{+/D9}$ and wild-type siblings. *Trim28* was equally expressed in the lean versus obese sub-groups (Figure S1A). Obese-Trim28$^{+/D9}$ animals exhibited increased adipose tissue mass in all measured depots including epididymal, inguinal, and brown fat pads (Figures 1D and 1E) and showed no observable difference in brain, kidney, spleen, muscle, or liver weights, indicating that bi-stability was at least partially restricted to adiposity. Documenting many individuals and litters, we observed that the Trim28$^{+/D9}$-sensitized obesity emerged during a relatively short window in early adulthood (8–12 weeks of age; Figure 1F) and then remained stable. Of potential interest, non-significant trends toward increased body mass and length were also observed in lean-Trim28$^{+/D9}$ relative to wild-type siblings (Figures 1A–1F).

Notably, inheritance of obesity was non-Mendelian. To date, the bi-stability phenotype has survived >7 years through breeding schemes including maternal and paternal transmission of the Trim28$^{+/D9}$ allele, as well as extensive backcrossing (> N10), inbreeding (> F20), three different mouse houses (QIMR, Australia; IMBA, Austria; and MPI-IE, Germany; Figure 1G), and embryo transfer. Comparison of interquartile ranges, coefficient of variation, and standard deviation against large, independently obtained non-early-life-controlled cohorts confirmed the heightened variation specifically of the Trim28$^{+/D9}$ heterozygotes (Figure S1B). We have observed average facility-specific obesity-"on" rates ranging from as low as ~5%–20% (MPI-IE, Freiburg) to nearly ~50% (QIMR, Australia) of the heterozygote Trim28$^{+/D9}$ population, suggesting that external factors can reproducibly influence the on/off decision. Though periods of apparent sex bias have been observed, the phenotype has been observed in both male and female animals and, as mentioned above, passes through both the male and female germlines. Importantly, the non-Mendelian bi-stability has survived dedicated attempts to segregate it; obese-Trim28$^{+/D9}$ fathers show no evidence of producing more obese offspring relative to their lean-Trim28$^{+/D9}$ siblings.

Importantly, conditional homozygous deletion of *Trim28* in muscle (Mck-Cre), adipose (Adipoq-Cre), liver (Alb-Cre), and

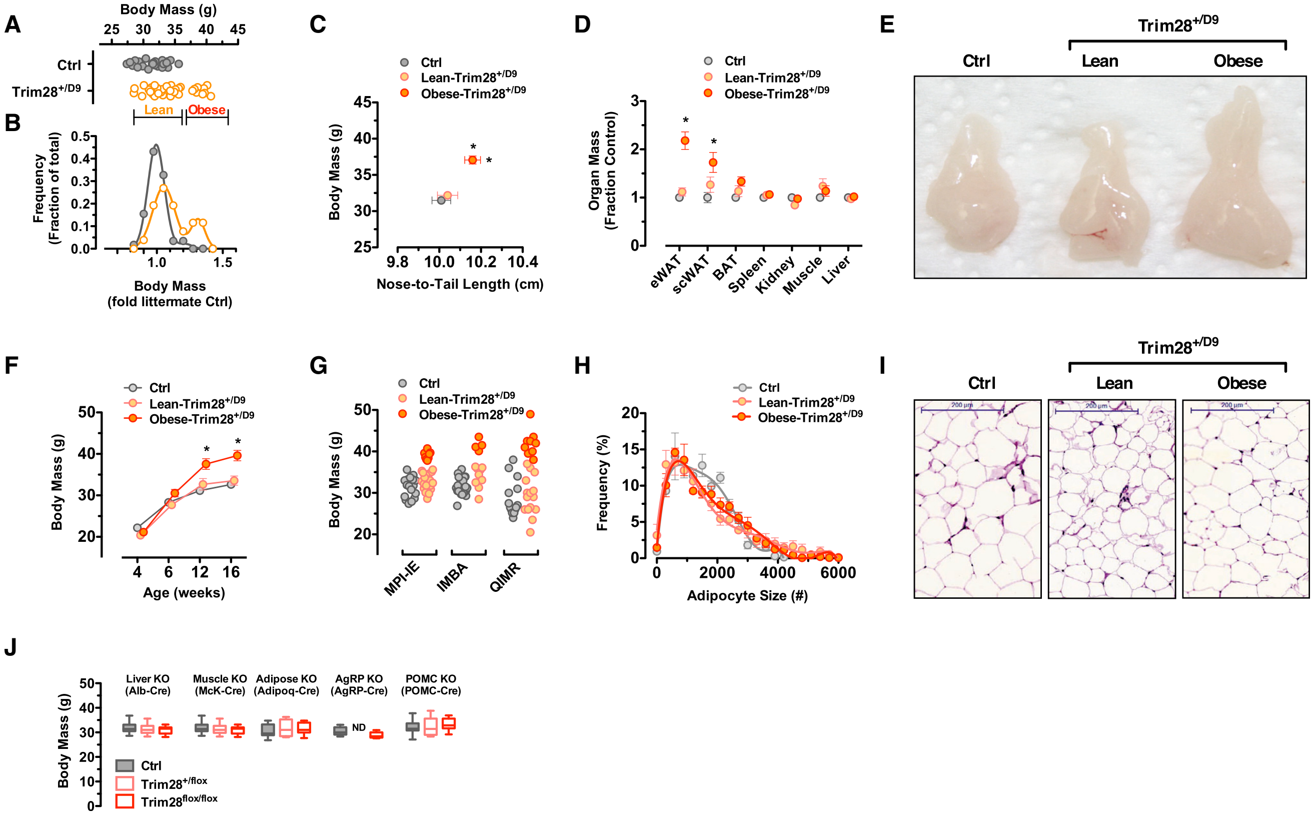

Figure 1. Trim28 Haploinsufficiency Induces Stochastic, Bi-stable Obesity in the Mouse

(A and B) Body mass of wild-type littermates and Trim28$^{+/D9}$ mice at 14–18 weeks of age (A) and frequency distribution of body weight normalized to wild-type littermates (B).

(C) Obese-Trim28$^{+/D9}$ mice are heavier and longer relative to wild-type and lean-Trim28$^{+/D9}$ mice.

(D and E) Increased mass in obese-Trim28$^{+/D9}$ results from expansion of fat depots (eWAT, scWAT, and BAT) and not organomegaly.

(F) Trim28$^{+/D9}$ mice are lighter at weaning relative to wild-type littermates; body-weight differences between lean- and obese-Trim28$^{+/D9}$ mice emerge near adulthood.

(G) The Trim28$^{+/D9}$ colony body-mass distributions at three different sites (MPI-IE, Germany; IMBA, Austria; QIMR, Australia) with a variable frequency ranging from 10%–50%.

(H and I) H&E staining of epididymal adipose (scale bar: 200 μm) shows no sign of adipocyte hypertrophy in Trim28$^{+/D9}$ mice.

(J) Tissue-specific knockout of *Trim28* in liver (Alb-Cre), muscle (McK-Cre), adipose (Adipoq-Cre), POMC (POMC-Cre), or AgRP (AgRP-Cre) neurons does not impact body mass.

Data are mean ± SEM (*$p < 0.05$). See also Figure S1.

the satiety regulating POMC- (POMC-Cre), and AgRP-neurons (AgRP-Cre) have revealed no obvious effect on adiposity (Figure 1J). These data indicate that TRIM28 is largely dispensable in fully differentiated adult tissues and support a role, consistent with much literature, in transcriptional programming in development. Thus, Trim28$^{+/D9}$ mice exhibit non-Mendelian bi-stable obesity.

Obese-Trim28$^{+/D9}$ Animals Are Metabolically “Healthy”

To better understand the adiposity phenotype, we metabolically phenotyped cohorts of Trim28$^{+/D9}$ animals. Examining oral glucose tolerance (Figure S1C), serum-free fatty acids, triglycerides (Figures S1D and S1E), as well as fasting glucose and insulin levels (Figures S1F and S1G), we found no evidence of major anomalies in the obese-Trim28$^{+/D9}$ animals nor in all Trim28$^{+/D9}$ animals grouped as a whole. Key determinants of metabolic health in obesity include efficient adipocyte turnover and function, as well as resistance to chronic metabolic inflammation (Gregor et al., 2013; Teperino et al., 2012; Jais et al., 2014). Consistent with the relatively protected metabolic phenotype, obese-Trim28$^{+/D9}$ animals showed no significant changes in adipocyte size (Figures 1H and 1I). Given the measured white adipose tissue mass differences (Figure 1D), the data are consistent with an approximate doubling of adipocyte number in the obese-Trim28$^{+/D9}$ animals. Plasma levels of the pro-inflammatory adipokines TNF-α and resistin, of C-reactive protein (Ouchi et al., 2011), and of the plasma-soluble receptor RAGE (Alexiou et al., 2010) were unremarkable (Figure S1H). Further, no evidence was found of secondary metabolic complications such as hepatosteatosis (Figure S1I), though our own previous work examining older cohorts has shown this as a potential endpoint (Whitelaw et al., 2010). mRNA expression profiling of epididymal

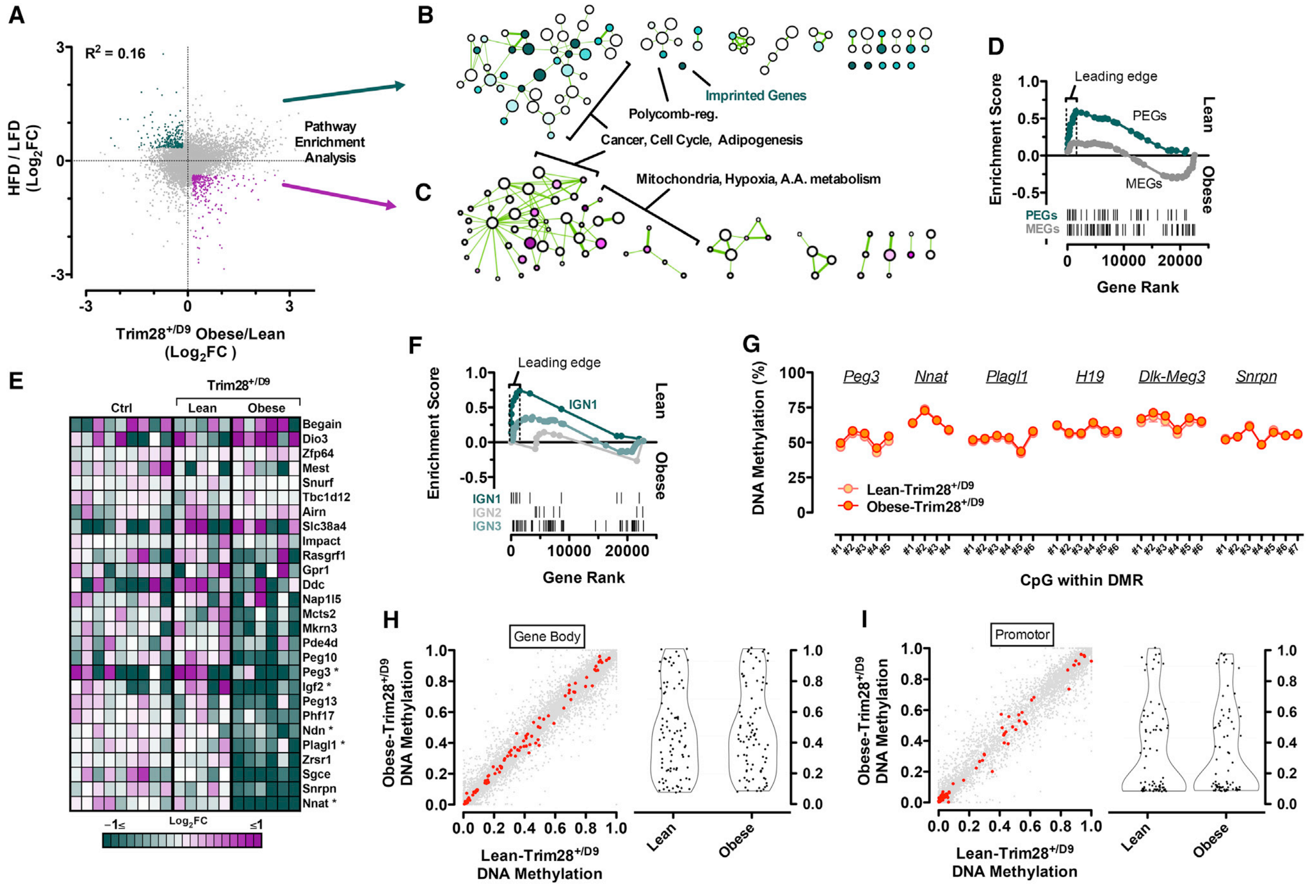

Figure 2. Non-classical Imprinted Gene Dysregulation Specifies the Obesity "On" State

(A–C) Poor correlation between measures of Trim28$^{+/D9}$-sensitized obesity and diet-induced obesity (high-fat diet and low-fat diet; HFD/LFD; GSE38337). Anti-correlated genesets from the transcriptome comparison (A) underwent MsigDb pathway enrichment analysis to reveal downregulated (B) or upregulated (C) pathway enrichment specific to obese-Trim28$^{+/D9}$ adipose.

(D) GSEA analysis reveals marked PEGs downregulation specifically in obese-Trim28$^{+/D9}$ mice.

(E) Heatmap visualization of the same RNA-seq data reveals that a subset of expressed PEGs (FPKM > 0.3) is downregulated in obese-Trim28$^{+/D9}$. Genes marked with an asterisk belong to IGN1. Columns represent sequenced biological replicates.

(F) GSEA enrichment of imprinted gene networks (IGN1-3; Al Adhami et al., 2015).

(G–I) Imprinted genes are dysregulated non-classically. (G) No changes in DNA methylation at germline DMRs measured by quantitative bisulfite and (H) no changes at the gene body or (I) promoters of imprinted genes as measured by RRBS in mature adipocytes. Imprinted genes are shown in red. RRBS data represent the mean of two independent replicates per group.

Data are mean ± SEM (*$p < 0.05$). See also Figure S2 and Tables S1, S2, S3, and S4.

adipose by RNA sequencing revealed moderate expression changes associated with both positive (elevated *Fgf21*, *Fndc5*; decreased *Retn*; Figure S1J) and negative metabolic outcomes (increased *Lep*, *Tnfa*, *IL1b*, and *Hmox1*; Figure S1K), indicating that although largely normal, the animals are likely not immune to metabolic complication.

Characterizing energy homeostasis has been difficult. Specifically, anxious and stress-sensitive behavior of the Trim28$^{+/D9}$ line has restricted successful analysis to a select few individuals despite use of stress-limiting home-cage indirect calorimetry. The data indicate no changes in food intake, expected alterations in V_{O2} and V_{CO2}, as well as a tendency toward reduced activity (Figures S1L–S1O). Statistical detection of differences in energy expenditure will require a high sampling number, which, given the stochasticity and stress sensitivity of the model, is an ongoing challenge.

Non-classical Imprinted Gene Dysregulation Specifies the Obesity "On" State

To find causal underpinnings for the obesity induction, we intersected RNA-sequencing datasets comparing epididymal adipose tissue gene expression in our obese- and lean-Trim28$^{+/D9}$ animals with comparable data contrasting high-fat diet (HFD)-induced obese animals with chow-fed controls (GSE38337). Global analysis of the two differential obesity datasets revealed low overall correlation (Pearson R = 0.16; Spearman R = 0.11; Figure 2A), indicating that the Trim28$^{+/D9}$-induced obese state is distinct from that induced by over-feeding.

Because Trim28$^{+/D9}$ was originally identified as a silencing "E(var)" mutation (Whitelaw et al., 2010), we focused on genes downregulated in obese-Trim28$^{+/D9}$ animals but unaltered or increased in HFD-induced obesity. Querying the top 250 such genes against the MSigDB collection of curated gene pathway annotations, we observed enrichment in genesets associated with proliferation/cell cycle/cancer, with adipogenesis/stem cell differentiation, and interestingly with Polycomb and HDAC-associated chromatin regulation (Figures 2B and 2C and Tables S1, S2, and S3).

One of the most enriched signals, top among chromatin-related pathways, was the geneset "Brideau_Imprinted_Genes" (Brideau et al., 2010) (Figures 2B and S2C). Imprinted genes comprise ~150 genomic loci whose expression follows a DNA methylation-associated parental-origin pattern, such that only one of the two alleles, either the maternally or paternally inherited one, is expressed. We built custom pathway annotations comprising all known paternally and maternally expressed imprinted genes (PEGs and MEGs, respectively) and probed for enrichment in our RNA-seq data using gene set enrichment analysis (GSEA). Whereas MEGs as a group exhibited less remarkable regulation, PEGs showed marked pathway downregulation specifically in obese-Trim28$^{+/D9}$ samples (Figures 2D, 2E, and S2D). Heatmap and GSEA enrichment score profiles suggested that the pathway contained two roughly equally sized gene subsets: one, prominently downregulated ("leading edge"; Figures 2D and 2E and Table S4), and a second, apparently non-regulated, subset with random distribution. Al Adhami et al. recently used an unbiased in silico approach to show that imprinted genes cluster into at least three co-regulated groups, suggesting functional compartmentalization beyond paternal and maternal expression (Al Adhami et al., 2015). Querying our data against these non-classical "imprinted gene network" (IGN) annotations at the pathway level, we found almost exclusive regulation of IGN1, a predominantly paternally expressed cluster previously implicated in body size/weight control (Varrault et al., 2006; Gabory et al., 2009; Al Adhami et al., 2015) (Figure 2F). The core enrichment signal of this analysis was driven by downregulation of *Plagl1, Dlk1, Cdkn1c, Nnat, Igf2, Peg3, Ppp1r9a, Ndn,* and *Grb10* in obese-Trim28$^{+/D9}$ animals (Figure 2F and Table S4). The downregulation of *Nnat*, *Plagl1*, and *Peg3* was confirmed by qPCR and at the protein level for *Nnat* (Figures S3E and S3F). Comparable regulation was not observed in data from HFD-induced obese adipose tissue (Figure S2G). Thus, an IGN1-centric gene signature characterizes Trim28$^{+/D9}$-induced obesity.

Mechanistically, imprinting results from discordant maternal versus paternal DNA methylation patterning at germline-defined imprinting control regions (ICRs). We next examined DNA methylation levels at IGN1 ICRs. Included were ICRs of three of our most differentially regulated IGN1 genes, including two moderately (*Plagl1*, *Peg3*) and one highly expressed (*Nnat*) gene as well as ICRs of unaffected imprinted loci within (*H19* and *Dlk1-Meg3*) and outside (*Snrpn*) the IGN1 network. Changes in *H19* and *Snrpn* have previously been linked to *Trim28* dysregulation in vivo (Messerschmidt et al., 2012). Importantly, we found no evidence of altered DNA methylation at any of these loci (Figure 2G). These findings indicated intact imprinting control and were true for assessments of both purified white epididymal adipocytes (Figure 2G) and stromal vascular preparations including adipocyte progenitor cells and pre-adipocytes (Figure S2H). Extension "genome-wide" using reduced representation bisulfite sequencing (RRBS) provided no evidence of significant changes in DNA methylation also beyond ICRs, including at promoters or gene bodies (Figures 2H and 2I). These findings were consistent with a lack of DMR-associated reciprocal gene-expression patterns that would be expected from classical loss of imprinting. Thus, a non-classical imprinted gene signature specifies Trim28$^{+/D9}$-dependent bi-stable obesity.

IGN1 Perturbation Induces Bi-stable Obesity

To test whether IGN1 dysregulation could cause obesity, we examined the phenotypic outcomes of targeted deletion of relevant IGN1 genes. We used Gateway-based "knockout-first" conditional targeting of exons 2 and 3 in JM8.F6 C57Bl6/N embryonic stem cells (ESCs) according to standard EUCOMM protocols to generate a knockout allele of our most strongly dysregulated IGN1 gene, *Nnat* (Figure 3A). ESCs were injected into blastocysts and implanted into pseudo-pregnant females, and chimeric offspring were screened for germline transmission. Deletion was confirmed on the RNA and protein levels (Figures 3B and 3C), and the newly derived Nnat knockout line was backcrossed more than ten generations to C57Bl6/J.

Nnat mutant offspring of both maternal and paternal transmission were born at Mendelian ratios. In support of a causal role for *Nnat* downregulation in Trim28$^{+/D9}$-induced obesity and bi-stability, paternal deletion mutants (Nnat$^{+/-p}$) exhibited a hyper-variable adiposity phenotype, again clearly emerging in adulthood (Figure 3D). Obese individuals were observed in both male and female offspring, and consistent with *Nnat* being paternally expressed, obesity was only observed upon paternal inheritance of the deleted allele. No evidence of obesity was detected in either wild-type littermates or cohorts receiving the imprinted deletion allele from their mothers (Nnat$^{+/-m}$; i.e., normal paternal *Nnat* expression) (Figure 3D). Fitting the body-weight distribution to the sum of two Gaussians (R^2 = 0.98) indicated an obesity-on rate of ~26% of the mutant population, with a 40% increase in body weight between the obese- and lean-Nnat$^{+/-p}$ animals (Figure 3E). Similar to the Trim28$^{+/D9}$ line, the bi-stable phenotype has been confirmed in more than one facility (MRC Cambridge and MPI-IE Freiburg; Figure 3F) and has survived embryo transfer and backcrossing onto pure C57Bl6/J as well as mixed C57Bl6/J:FVBN/J F1 backgrounds. Obesity in Nnat$^{+/-p}$ animals was characterized by increased adipose tissue mass in epididymal adipose pads and on the whole animal level by dual-emission X-ray absorption analysis (Figures 3G and S3A). These findings implicate *Nnat* in buffering non-Mendelian bi-stable adiposity.

We also found evidence of potential bi-stability in an additional IGN1-targeted mouse model, a conventional knockout of the paternally expressed *Peg3* allele (Peg3$^{+/-p}$). Curley et al. reported increased body-fat accumulation in both male and female Peg3$^{+/-p}$ mutants (Curley et al., 2005). Re-analysis of those data revealed a population-level fat-mass distribution consistent with two adiposity-discordant sub-populations with an estimated obesity-on rate of ~20% and a near-doubling in body

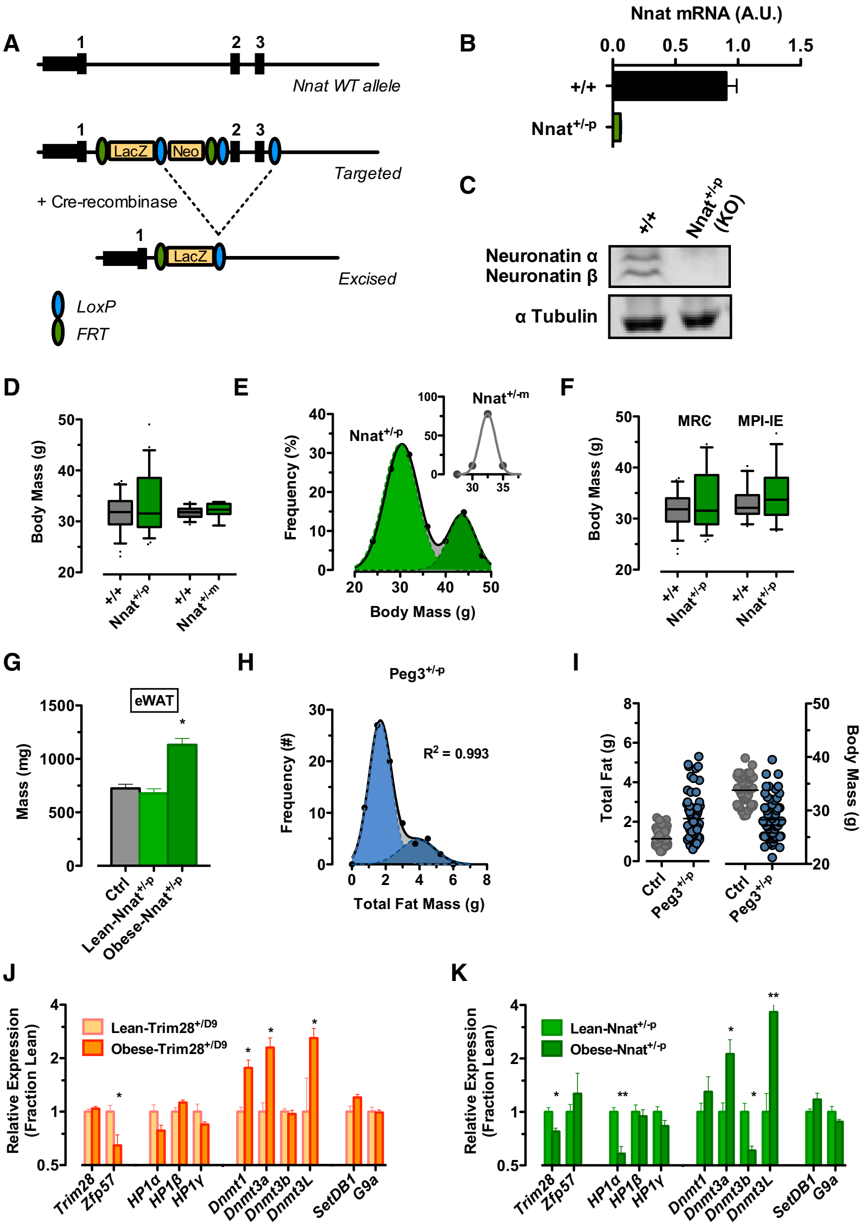

Figure 3. *Nnat* and *Peg3* Knockout Mice Exhibit Bi-stable Obesity

(A) Targeting approach for a "knockout first" *Nnat* deletion allele.

(B) mRNA and (C) protein-level expression in E17.5 embryo heads of paternal *Nnat* null (Nnat$^{+/-p}$) mutants and their wild-type littermate controls (+/+).

(D) Hypervariable body mass at 14–18 weeks of age observed upon deletion of the paternal and not maternal (Nnat$^{+/-m}$) allele.

(E) Body-mass distribution is bi-modal (gray). The single-Gaussian sub-distributions of the double-Gaussian fit (gray) are shown in green. Inset highlights that body-mass distribution upon maternal transmission (Nnat$^{+/-m}$) of the Nnat null allele follows a single-Gaussian distribution.

(F) Hypervariable body-mass distributions observed at two different sites (MPI-IE, Germany; MRC, UK). Shown are male progeny of ~10 litters at each site.

(G) Epididymal adipose tissue mass from lean- and obese-Nnat$^{+/-p}$ mice and their littermate controls.

(H) Body-fat distribution of Peg3$^{+/-p}$ mice is bi-modal (gray). The single-Gaussian sub-distributions of the double-Gaussian fit (gray) are shown in blue (re-graphed from Curley et al., 2005).

(I) Individual replicates for total fat mass and body weight for Peg3$^{+/-p}$ mice (re-graphed from Curley et al., 2005).

(J and K) Obese-Trim28$^{+/D9}$ and obese-Nnat$^{+/-p}$ animals exhibit decreased mRNA expression of most of the recruitment factors (*Zfp57*, *Hp1α*, and *Hp1γ*) concomitant with increased expression of silencing factors *SetDb1*, *Dnmt's 1*, *3a*, and *3L* relative to their lean siblings.

Data are median with interquartile range (boxplots) or mean ± SEM (*$p < 0.05$). See also Figure S3.

fat between lean and obese mutants (N = 80) (Figure 3H). Interestingly, Peg3$^{+/-p}$ mice were reported to be uniformly smaller, with concomitant reductions in body size, weight, and skeletal muscle mass, indicating that gene dosage among IGN1 members might be important for phenotypes beyond adiposity (Figure 3I). To the best of our knowledge the *Peg3* deletion strain was not maintained by the community. We are now in the process of backcrossing a re-derived line from the original targeting construct.

During development, TRIM28 controls endogenous retroviruses and germline ICRs in the context of TRIM28-ZFP complexes. Hoping for additional mechanistic insight into the obesity decision, we also mapped our RNA-seq reads to a compendium of genomic repeat sequences as TRIM28 has previously been implicated in control of repeat expression (Rowe et al., 2010). Examining ERV families and classes and uniquely mapping reads and chimeric reads between genes and transposable element regions, we found few significant changes. Indeed, the number and degree of repeat expression alterations were lower than in comparable mutants of cellular metabolism (data not shown). Interestingly though, when examining RNA expression of the imprint-regulating TRIM28-ZFP57 complex, and related genes, in adipose of the Trim28 and Nnat mutant lines, we observed consistent dysregulation. Obese-Trim28$^{+/D9}$ animals exhibited decreased expression of the guide/recruitment factors *Zfp57*, *Hp1α*, and *Hp1γ*, concomitant with increased expression of *Dnmt1*, *3a*, and *3L* relative to their lean-Trim28$^{+/D9}$ siblings (Figure 3J). The same trend was observed in Nnat$^{+/-p}$ animals, except that instead of *Zfp57*, *Trim28* was reduced in obese individuals (Figure 3K). These data are consistent with a model wherein relative dosage of TRIM28-ZFP57 complex recruitment and silencing functions underpin the switch between lean and obese phenotypes.

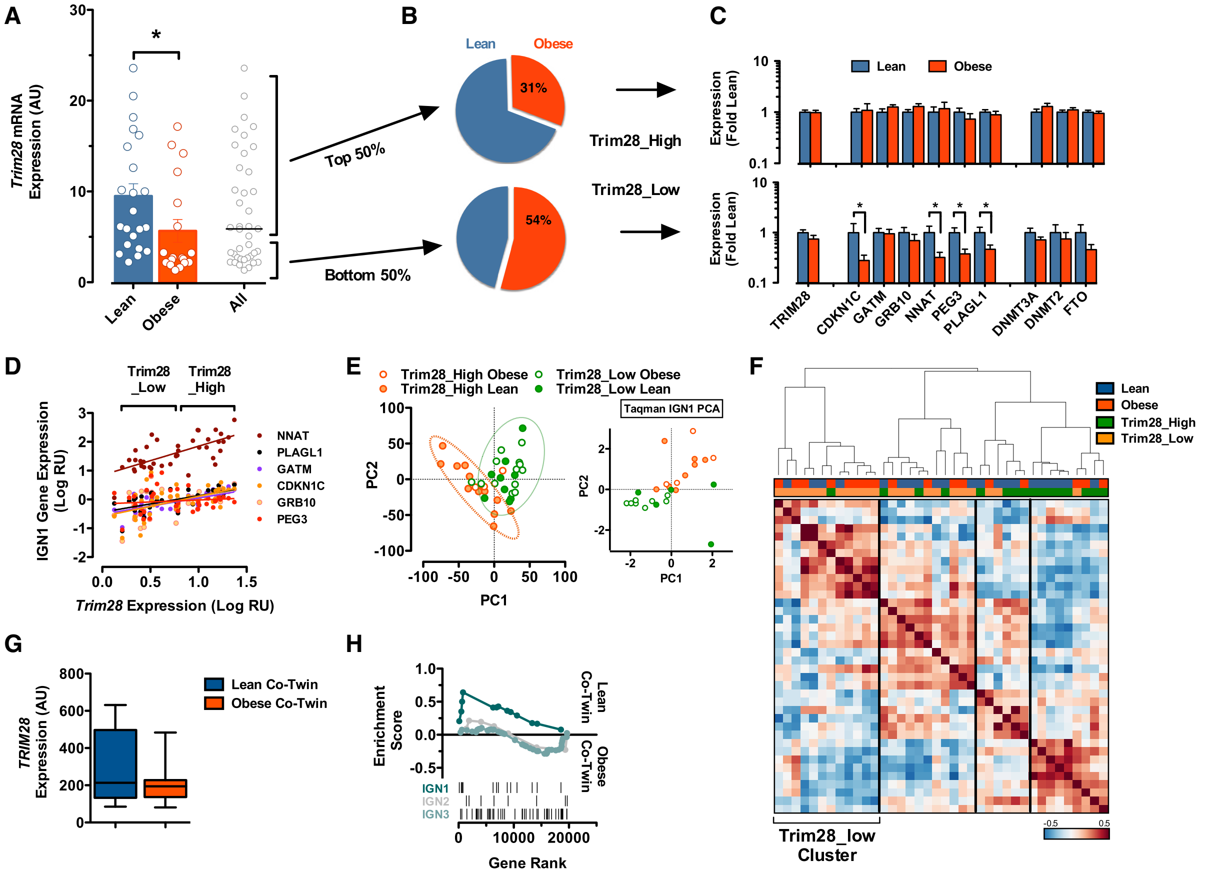

Figure 4. Trim28-Low Human Children Are Obesity Susceptible and Exhibit a Distinct Transcriptome Landscape

(A) Taqman qPCR of *TRIM28* mRNA expression in sub-cutaneous adipose from human pre-pubescent children.

(B) Stratification by BMI indicates that the obese sub-group is enriched for Trim28-low individuals.

(C) Taqman qPCR shows that obese, Trim28-low individuals specifically exhibit reduced expression of IGN1-imprinted genes.

(D) Correlation of *TRIM28* versus IGN1 member gene expression.

(E) PCA of adipose tissue RNA-seq from the same individuals reveals Tim28-low versus -high individuals to be substantially different. Inset highlights the same to be true when analyzing only IGN1 qPCR data.

(F) Heatmap visualization of hierarchical clustering of the most variable 6,000 genes expressed in adipose from the cohort. Vertical lines are for visualization purposes only.

(G) *TRIM28* and (H) IGN1 pathway expression are selectively decreased in obese co-twins in a cohort of 13 discordant monozygotic twin pairs (Pietiläinen et al., 2008).

Data are mean ± SEM (*p < 0.05) or min-to-max whiskers. See also Figure S4.

Evidence of TRIM28-Associated Phenotypic Bi-potential (Polyphenism) in Humans

We reasoned that if present, evidence of bi-stability and Trim28/IGN1-associated obesity would be most evident in childhood because this represents a window of tight environmental control in humans (i.e., essentially all children in the developed world exhibit largely coincident and enforced circadian, feeding, and activity patterns imposed through parenting, primary care, and education). We examined gene expression by Taqman qPCR in subcutaneous adipose tissue samples from a cohort of pre-pubertal Caucasian children of European ancestry entering the clinic for elective surgery (typically orthopedic); the cohort included 22 lean and 18 obese individuals. The children were disease and medication free and have been described in detail as part of the Leipzig Childhood AT cohort (Landgraf et al., 2015). When measured against three housekeeping genes (*ACTB*, *HPRT*, *TBP*), we found both a significant reduction in adipose tissue *TRIM28* levels in obese children (Figure 4A) and an apparent cluster of very low *TRIM28*-expressing individuals in the obese group. Individuals in the lower 50th percentile of *TRIM28* expression appeared more likely to be obese than high *TRIM28* expressors (Figure 4B). Mimicking analysis of the Trim28$^{+/D9}$ haploinsufficient mouse scenario, we sub-divided all individuals into obese or lean groups of comparably high or low *TRIM28* levels (Trim28_High and Trim28_Low, respectively) and used qPCR to measure expression of six of our leading-edge, Trim28$^{+/D9}$-obesity-associated IGN1 genes (Figures 4C and 4D). IGN1 genes correlated with *TRIM28* expression (Figure 4D). Whereas Trim28_High individuals showed essentially identical IGN1 expression levels irrespective of adiposity (Figure 4C, upper

panel), obese Trim28_Low subjects showed clear reductions in *CDKN1C*, *PLAGL1*, *NNAT*, and *PEG3* expression when compared to equally Trim28_Low lean subjects (Figure 4C, lower panel). These data recapitulate our observations of enhanced IGN1 downregulation in $Trim28^{+/D9}$ haploinsufficient mice and are consistent with a conserved role for a Trim28-IGN1 axis in human adiposity regulation.

To test whether the observations reflected differences beyond IGN1 output, we performed RNA sequencing of the same adipose samples and analyzed transcriptome organization with principal-component analysis (PCA) of the 6,000 most variable, expressed genes. The PCA revealed a non-homogeneous population distribution with two apparent sub-populations (Figure 4E). Plotting individuals according to *TRIM28* expression revealed that the clusters did not discriminate obese from lean or male from female subjects (Figures S4A and S4B) but rather Trim28_High from Trim28_Low individuals (Figure 4E). These findings recapitulate PCA analysis of the IGN1 gene qPCR data from the same individuals (insets, Figures 4E, S4A, and S4B) and indicated that on the transcriptome level Trim28_High and _Low individuals are more different than lean versus obese or male versus female individuals. These patterns were equally clear when assessing the data in a Pearson correlation matrix (Figure 4F) and by hierarchical clustering (Figure S4C). Thus, humans appear to stratify into sub-populations defined by adipose *TRIM28* expression, with Trim28_Low individuals exhibiting distinct transcriptional complexity, IGN1 dysregulation, and increased obesity incidence.

BMI Bi-modality at the Population Level in Humans

Next, we examined publically available datasets for signs of bimodal body-weight distributions in the general population. We first examined adipose tissue microarray data from 13 discordant monozygotic (MZ) twin pairs each comprising one obese and one normal co-twin (Pietiläinen et al., 2008). Importantly, we found suggestions of both reduced mean *TRIM28* levels (Figure 4G) and reduced IGN1 pathway expression (GSEA; Figure 4H) specifically in obese relative to lean isogenic co-twins. This indicates that Trim28-IGN1 expression correlates with epigenetically rooted human obesity.

We analyzed BMI distributions of ~4,000 Caucasian children, 6–11 years old, surveyed by the National Health and Nutrition Examination Survey (NHANES) between 1999 and 2012 (Center for Disease Control) (CDC, 2012). As is well described for the general population, BMI distributions were positively "skewed." We observed, however, that this positive bias was not gradual but rather contained a distinct inflection point suggestive of a mixed distribution (Figure 5A). We noted during the analysis that bar-chart visualization, as well as large and/or irregular bin-sizings often used in BMI analysis, mask this inflection. Importantly, where a single Gaussian failed to accurately model the data (and in particular the positive skew), we found that a mixed model assuming two independent and potentially overlapping Gaussian sub-populations fit ~99.2% of the data distribution (R^2 = 0.99; sum-of-two-Gaussians: R^2 = 0.91; single Gaussians) (Figures 5A and 5B). Inspection on a log-scale revealed that only the most obese children (> ~30 BMI; <1% of the population) fell outside confidence intervals (Figures S5A and 5B). Of note, analyses compensating for the complex NHANES sampling strategy provided equivalent results. Most importantly, we observed the same pattern and goodness-of-fit when independently analyzing BMI distributions of males and females, of Mexican American, Hispanic, Caucasian, and African American children (Figure 5C), as well as of adult populations examined inside and outside North America, including a cohort of ~10,000 Han Chinese individuals (Figures 5D and 5E).

Finally, we found evidence of a marked frequency transition between sub-populations over recent decades. We compared BMI distributions from NHANES data gathered between 1963 and 1994 (CDC, 1994) with the more recent 1999–2012 (continuous NHANES) data (CDC, 2012). Both datasets followed a sum-of-two-Gaussians fit with an R^2 > 0.99. In contrast to the popularized notion that the population as a whole is significantly gaining weight, we observed that the calculated mean BMI of the major (lean) population increased only 0.07 BMI units from 1963–1994 (BMI = 15.81; males 6–11 years of age) to 1999–2012 (BMI = 15.87), i.e., +0.4%. Instead, the profiles indicated an increase in the relative fraction of individuals falling into the second (heavier) sub-population. The heavy sub-population, with a mean BMI 4–5 points above "normal," more than tripled, from ~12% to ~38% of all individuals. These findings agree with other descriptions of childhood obesity (Körner et al., 2007) and suggest increased incidence of a distinct category of "triggered" individuals that would be consistent with the notion of polyphenism. Given that mean normalizations do not effectively accommodate variable bimodality, these data suggest that normalizations to age- and sex-specific medians, modes, or Winsorized means may more accurately align population-level BMI variation.

DISCUSSION

Phenotypic variation describes the extent and character of variability in a given phenotype in the population and is thought to be a platform for adaptation and evolution. It can be of genetic or epigenetic origin, or both (Simpson et al., 2011). Polyphenism refers specifically to the case where individuals of the same genotype can exhibit multiple discrete phenotypic end-points without intermediates and has been most heavily studied in insects. Classical examples include seasonal morphs in butterflies, caste morphs in eusocial insects (worker, soldier, queen), and the intriguing intergenerational reproductive morphs of aphids. The observation of two distinct phenotypic end-points in isogenic Trim28-, Nnat- and possibly Peg3- mutant mice indicates similar phenotypic bi-potential in mice and identifies a genetic network that buffers against emergence of divergent states.

Although unclear whether socially, genetically, or epigenetically underpinned, the relatively inbred wild mole-rat exhibits a somewhat parallel dispersal morph that arises in times of plenty, exhibits increased adipose tissue mass, and shows a behavioral phenotype that ultimately leads it to leave the colony (O'Riain et al., 1996). The uncommon phenotype is thought to provide populations with the cooperative advantages of eusocial behavior while avoiding complete inbreeding. Similarly, increased energy storage induced via the $Trim28^{+/D9}$-sensitive axis described here might be expected to protect from predation

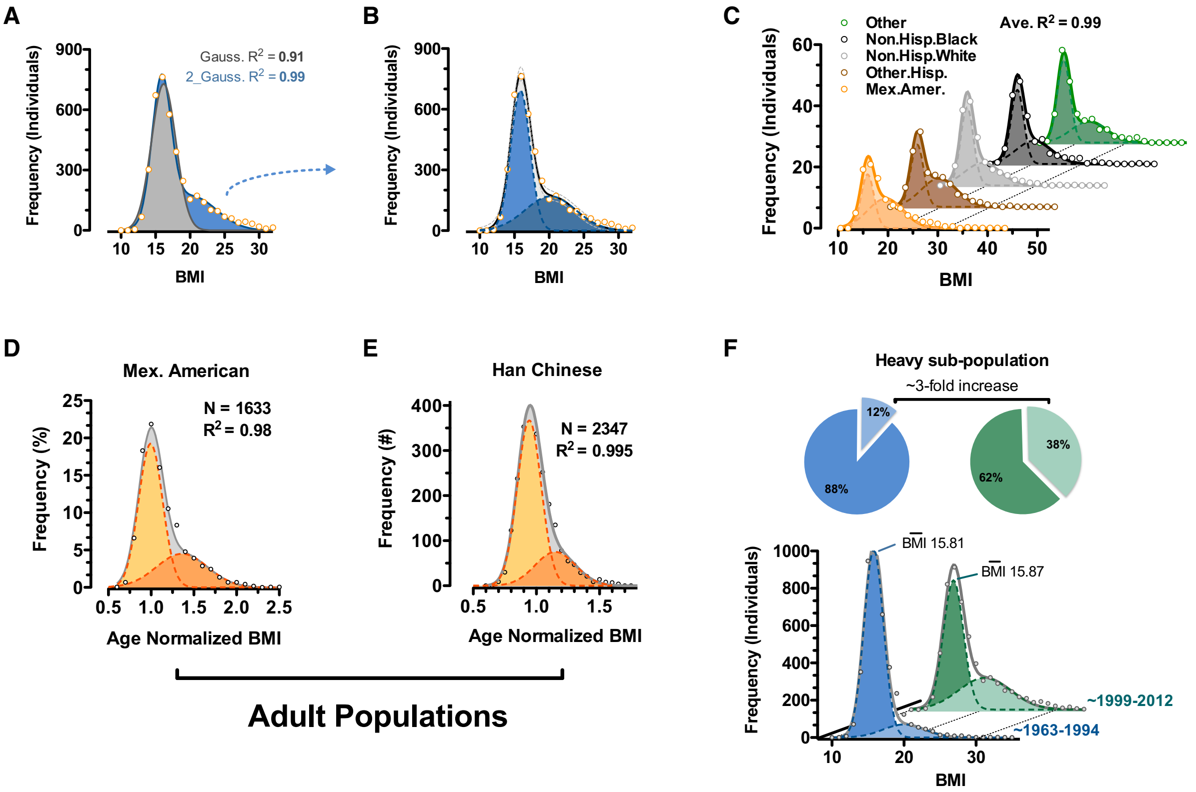

Figure 5. BMI of the General Population Is Consistent with a Bi-modal Distribution

(A) BMI distribution of 6- to 11-year-old non-hispanic white males from the continuous NHANES 1999–2012 survey (CDC, 2012). Data are fit to a single Gaussian (gray) and a double Gaussian (blue).

(B) Individual Gaussian components of the double Gaussian from (A).

(C) Near-perfect double-Gaussian fit is observed across children of five major ethnicity classes, as well as in adult cohorts from (D) continuous NHANES 1999–2012 (CDC, 2012) and (E) Han chinese populations.

(D and E) Shown are age-normalized BMI distributions for females aged 25–50.

(F) Comparison of similar fitting of childhood data from continuous NHANES 1999–2012 (CDC, 2012) and prior NHANES/NHES surveys (1963–1994) (CDC, 1994) shows a marked shift in recent decades where the heavy sub-population triples in size (pie charts).

See also Figure S5.

and starvation. Although the nature of triggers for our observed obese state is not clear, we have observed extended periods with reduced obese individuals correlating with housing density and reduced temperature. At the site of generation of the Trim28$^{+/D9}$ line (QIMR, Australia), obese individuals were observed at higher rates; that facility was warmer but also had different microflora, rodent diet, and staff and is a non-SPF facility. Preliminary experiments examining maternal HFD effects in Trim28$^{+/D9}$ animals suggest possible increased obesity rates.

Polyphenisms can also be genetically influenced. Our first efforts to map the molecular switch at work reveal coordinate suppression of IGN1 members. The mutant data implicate *Trim28, Nnat*, and *Peg3* dosage and function in triggering bi-potential. Human genetic variants near *NNAT* associate in some populations with *NNAT* expression and with obesity, also in children (Vrang et al., 2010), and paternal transmission of obesity in F1 background crosses has been associated with *Peg3* variation (Morita et al., 2014). Together, these findings indicate multiple genetic entry points for sensitization of the described epigenetic obese state. The bi-modal fit of human population-level BMI distributions would suggest that genome-wide association study (GWAS) efforts performed to date might already contain substantial signals for comparable non-Mendelian obesity. Generating tools to stratify and filter these datasets for epigenetic versus genetically driven phenotype would markedly enhance their power. Noteworthy in that regard, we specifically observed trends toward reduced *FTO* expression in Trim28_Low obese children relative to their Trim28_Low lean counterparts (Figure 4D) and progressive decreases in our lean- and obese-Trim28$^{+/D9}$ mice, respectively (Figure S5C). FTO is the highest scoring GWAS variant for obesity in both adults and children and, notably, one of only a handful that associates with variation in phenotype as well as magnitude (Yang et al., 2012).

Non-classical IGN1 dysregulation was one of the strongest signatures distinguishing Trim28$^{+/D9}$ obesity from HFD-induced obesity in mice and was a predictor of high-dimensional transcriptome variation in childhood adipose tissue. Preliminary F1 epistasis examination crossing B6.Nnat$^{+/-p}$ and FVB/NJ.Trim28$^{+/D9}$

animals has yet to reveal any marked increase in obesity incidence and therefore suggests that *Nnat* and *Trim28* lie in the same genetic pathway. IGN1 genes have been implicated in placentation (Sekita et al., 2008), development, growth, and importantly metabolic control (see Cleaton et al., 2014 for review), and five of the nine IGN1 genes show energy homeostasis defects when deleted: *Peg3* (Curley et al., 2005), *Plagl1* (Kamiya et al., 2000), *Grb10* (Smith et al., 2007), *Dlk1* (Moon et al., 2002), and *Nnat* (this manuscript). TRIM28 loss has previously been linked to dysregulation of imprinted genes during development (Messerschmidt et al., 2012), and interestingly, DNMT3A, the methyltransferase in part responsible for proper imprint deposition, has also been tied to buffering phenotypic variability (Whitelaw et al., 2010). Also noteworthy, combined heterozygous mutation of the insulin and insulin-like growth factor 1 receptors leads to reduced expression of a subset of IGN1-overlapping imprinted genes (Boucher et al., 2014), suggesting insulin and IGF1 signaling as potential regulatory candidates for the phenotypes observed here.

Currently we cannot rule out largely on/off environmental signals (e.g., parental smoking) as direct effectors of bi-modality in the human data presented, nor can we rule out somatic mutations or Trim28-stabilized mutational hotspots and transposon activity as genetic underpinnings for the observations presented here. If substantiated, however, the impact of phenotypic bi-stability for humans is substantial. They include academic, ethical, and therapeutic aspects, and understanding the number, nature, and disease associations of possible states will be paramount. Trim28 mutant mice show increased cancer susceptibility (Herquel et al., 2011) as well as anxiety and behavioral phenotypes (Whitelaw et al., 2010; Jakobsson et al., 2008). Critical next steps for us will include testing whether comparable behavioral, cancer-prone, or alternate epigenetic trajectories exist in the sensitized contexts presented here and ultimately also in humans.

EXPERIMENTAL PROCEDURES

Generation of *Trim28* and *Nnat* Mutant Mice

The generation of Trim28$^{+/D9}$ has been described elsewhere (Blewitt et al., 2005). For information on the generation of *Trim28* tissue-specific knockout mice and of *Nnat* null mice, see the Supplemental Experimental Procedures. All mouse models are described in the Supplemental Experimental Procedures. Animals were kept on a 12 hr light/dark cycle with free access to food and water and housed in accordance with international guidelines.

Human Study Population

Subcutaneous adipose tissue samples were obtained from 18 obese and 22 lean subjects of the Leipzig Childhood AT cohort (NCT02208141) (Landgraf et al., 2015). Children were 2–15 years old and included if they were in pre-puberty. For further information, see the Supplemental Experimental Procedures.

Glucose Tolerance Test

Oral glucose (1 g/kg) tolerance test was performed on 25-week-old animals as described in the Supplemental Experimental Procedures.

Indirect Calorimetry

To measure basal metabolic rate, 10- to 14-week-old animals were singly housed in a home-cage indirect calorimetry system (TSE Systems). Animals were monitored over a 6 day period and fed an ad libitum chow diet. Data from the first day were discarded to reduce variation introduced by acclimatization. Data from consecutive days were treated as technical replicates, and data were binned in 2 hr intervals. Food consumption was measured directly as accumulated data.

Mouse Laboratory Parameters and Cytokines

Fasted (6 hr fast) blood plasma were obtained, and a panel of hormones, fatty acids, and adipokines were measured as described in the Supplemental Experimental Procedures.

Histology, Immunofluorescence, Adipocyte Size, and Number

We used semi-automated morphometry with H&E-stained paraffin sections of perigonadal WAT pads to perform adipocyte number and size analyses. Images were analyzed using ImageJ. Immunofluorescence was performed as described in the Supplemental Experimental Procedures.

Isolation of Mouse Primary White Pre-adipocytes and Mature Adipocytes

Primary preadipocytes and mature adipocytes were obtained by collagenase digestion from perigonadal white adipose tissue in mice as described (Pospisilik et al., 2010).

qRT-PCR

For qRT-PCR of mouse and human samples, analysis of total RNA was performed on a 7900HT Fast Real-Time PCR System (Applied Biosystems). Mouse primers were designed using qPrimerDepot, and human "Best-Coverage" Taqman probes were purchased from Life Technologies. Threshold cycles (Ct-values) of all replicate analyses were normalized to *TBP* (mouse) or to *TBP*, *ACTB*, *HPRT* (human) housekeeping genes. To compare the effect of various conditions with controls, $2^{-\Delta\Delta Ct}$ values were calculated to obtain fold expression levels. For further information, see the Supplemental Experimental Procedures.

RNA Sequencing

Trizol-purified RNA was poly(A)-enriched, and libraries were prepared with a TruSeq Sample Prep v2 kit (Illumina) and sequenced on a HiSeq 2500 (Illumina). Greater than 15 million reads per mouse sample and 10 million reads per human sample were mapped using TopHat v2.0.8b with -G option against *Mus musculus* genome (mm9, iGenome UCSC) and *Homo sapiens* genome (hg19, iGenome UCSC), respectively. Gene expression values and significantly differentially expressed genes were calculated using Cuffnorm and Cuffdiff v2.2.1 with geometric normalization and multi-read correction (-u option).

Reduced Representative Bisulfite Sequencing

Total genomic DNA was digested over night with MspI (NEB), and sequencing libraries were prepared with NEBNext DNA Library kit (NEB). Samples were sequenced on a HiSeq 2500 (Illumina). For further information, see the Supplemental Experimental Procedures.

Bioinformatic Analyses

Gene set enrichment analysis used GSEA 2.0 with default parameters (permutation type: gene_set. Collapse dataset to gene symbols: false).

Statistical Analysis

Data are expressed as mean ± SEM unless otherwise specified. Statistical analyses were performed as described in the Supplemental Experimental Procedures. All animal experiments included at least four biological replicates, and all reported p values are two-tailed unless stated otherwise. $p < 0.05$ was used as a cutoff for statistical significance.

Other Methods

See Supplemental Experimental Procedures.

ACCESSION NUMBERS

Raw RNA-seq data from Trim28$^{+/D9}$ adipose tissue are available via the ENA, under project "PRJEB11740" (http://www.ebi.ac.uk/ena/data/view/PRJEB11740). Processed data are available in Tables S2 and S3.

SUPPLEMENTAL INFORMATION

Supplemental Information includes Supplemental Experimental Procedures, five figures, and five tables and can be found with this article online at http://dx.doi.org/10.1016/j.cell.2015.12.025.

AUTHOR CONTRIBUTIONS

Conceptualization, K.D. and J.A.P.; Methodology, K.D., J.A.P., A.C.F.-S., E.W., I.Z., and D.R.; Formal Analysis, Investigation, and Visualization, K.D., K.L., S.H., J.L., K.G., M.R., M.O., R.S., M.S., T.T.-H.L., E.C., R.T., I.Z., D.R., Y.C.L.T., B.L., R.L., and A.P.C.; Writing – Original Draft, K.D., J.A.P., and A.L.; Writing – Review & Editing, K.D., J.A.P., A.L., A.S., A.C.F.-S., A.P.C., and S.O.R.; Supervision, Resources, and Funding Acquisition, J.A.P., A.C.F.-S., T.V., J.M.P., T.J., C.-L.C., A.K., S.O.R., G.S.H.Y., and A.P.C.

ACKNOWLEDGMENTS

W. Reik, H. Esterbauer, F. Cammas, C. Imbusch, J.P. Curley, B. Kaverne, S. Smallwood, K. Nordstrom, K. Pietilainen, T. Manke, and L. Ancic for theoretical discussions, technical and analytical help and advice, and graphical inputs. This work was supported by funding from the Max-Planck Society, ERC (ERC-StG-281641), DFG (SFB992 "MedEp"; SFB 1052 "ObesityMechanisms"), EU_FP7 (NoE "Epigenesys"; "Beta-JUDO" n° 279153), BMBF (DEEP), MRC (Metabolic Disease Unit - APC, SOR, GSHY, MRC_MC_UU_12012/1), Wellcome Trust (SOR, 095515/Z/11/Z), and the German Research Council (DFG) for the Clinical Research Center "Obesity Mechanisms" CRC1052/1 C05 and the Federal Ministry of Education and Research, Germany, FKZ, 01EO1001 (Integrated Research and Treatment Center [IFB] Adiposity Diseases).

Received: July 30, 2015
Revised: October 12, 2015
Accepted: December 8, 2015
Published: January 28, 2016

REFERENCES

Al Adhami, H., Evano, B., Le Digarcher, A., Gueydan, C., Dubois, E., Parrinello, H., Dantec, C., Bouschet, T., Varrault, A., and Journot, L. (2015). A systems-level approach to parental genomic imprinting: the imprinted gene network includes extracellular matrix genes and regulates cell cycle exit and differentiation. Genome Res. *25*, 353–367.

Alexiou, P., Chatzopoulou, M., Pegklidou, K., and Demopoulos, V.J. (2010). RAGE: a multi-ligand receptor unveiling novel insights in health and disease. Curr. Med. Chem. *17*, 2232–2252.

Allshire, R.C., Javerzat, J.-P., Redhead, N.J., and Cranston, G. (1994). Position effect variegation at fission yeast centromeres. Cell *76*, 157–169.

Anway, M.D., Cupp, A.S., Uzumcu, M., and Skinner, M.K. (2005). Epigenetic transgenerational actions of endocrine disruptors and male fertility. Science *308*, 1466–1469.

Ashe, A., Sapetschnig, A., Weick, E.-M., Mitchell, J., Bagijn, M.P., Cording, A.C., Doebley, A.-L., Goldstein, L.D., Lehrbach, N.J., Le Pen, J., et al. (2012). piRNAs can trigger a multigenerational epigenetic memory in the germline of C. elegans. Cell *150*, 88–99.

Blewitt, M.E., Vickaryous, N.K., Hemley, S.J., Ashe, A., Bruxner, T.J., Preis, J.I., Arkell, R., and Whitelaw, E. (2005). An N-ethyl-N-nitrosourea screen for genes involved in variegation in the mouse. Proc. Natl. Acad. Sci. USA *102*, 7629–7634.

Boucher, J., Charalambous, M., Zarse, K., Mori, M.A., Kleinridders, A., Ristow, M., Ferguson-Smith, A.C., and Kahn, C.R. (2014). Insulin and insulin-like growth factor 1 receptors are required for normal expression of imprinted genes. Proc. Natl. Acad. Sci. USA *111*, 14512–14517.

Brideau, C.M., Eilertson, K.E., Hagarman, J.A., Bustamante, C.D., and Soloway, P.D. (2010). Successful computational prediction of novel imprinted genes from epigenomic features. Mol. Cell. Biol. *30*, 3357–3370.

Carone, B.R., Fauquier, L., Habib, N., Shea, J.M., Hart, C.E., Li, R., Bock, C., Li, C., Gu, H., Zamore, P.D., et al. (2010). Paternally induced transgenerational environmental reprogramming of metabolic gene expression in mammals. Cell *143*, 1084–1096.

Center for Disease Control and Prevention (CDC) (1994). National Center for Health Statistics (NCHS). National Health and Nutrition Examination Survey Data. Hyattsville, MD: U.S. Department of Health and Human Services, Centers for Disease Control and Prevention. Continuous NHANES Data 1963–1994. http://www.cdc.gov/nchs/nhanes/nhanes_questionnaires.htm.

Center for Disease Control and Prevention (CDC) (2012). National Center for Health Statistics (NCHS). National Health and Nutrition Examination Survey Data. Hyattsville, MD: U.S. Department of Health and Human Services, Centers for Disease Control and Prevention. Continuous NHANES Data 1999–2014. http://www.cdc.gov/nchs/nhanes/nhanes_questionnaires.htm.

Cleaton, M.A.M., Edwards, C.A., and Ferguson-Smith, A.C. (2014). Phenotypic outcomes of imprinted gene models in mice: elucidation of pre- and postnatal functions of imprinted genes. Annu. Rev. Genomics Hum. Genet. *15*, 93–126.

Curley, J.P., Pinnock, S.B., Dickson, S.L., Thresher, R., Miyoshi, N., Surani, M.A., and Keverne, E.B. (2005). Increased body fat in mice with a targeted mutation of the paternally expressed imprinted gene Peg3. FASEB J. *19*, 1302–1304.

Daxinger, L., and Whitelaw, E. (2012). Understanding transgenerational epigenetic inheritance via the gametes in mammals. Nat. Rev. Genet. *13*, 153–162.

Daxinger, L., Harten, S.K., Oey, H., Epp, T., Isbel, L., Huang, E., Whitelaw, N., Apedaile, A., Sorolla, A., Yong, J., et al. (2013). An ENU mutagenesis screen identifies novel and known genes involved in epigenetic processes in the mouse. Genome Biol. *14*, R96.

Gabory, A., Ripoche, M.-A., Le Digarcher, A., Watrin, F., Ziyyat, A., Forné, T., Jammes, H., Ainscough, J.F.X., Surani, M.A., Journot, L., and Dandolo, L. (2009). H19 acts as a trans regulator of the imprinted gene network controlling growth in mice. Development *136*, 3413–3421.

Gapp, K., Jawaid, A., Sarkies, P., Bohacek, J., Pelczar, P., Prados, J., Farinelli, L., Miska, E., and Mansuy, I.M. (2014). Implication of sperm RNAs in transgenerational inheritance of the effects of early trauma in mice. Nat. Neurosci. *17*, 667–669.

Greer, E.L., Maures, T.J., Ucar, D., Hauswirth, A.G., Mancini, E., Lim, J.P., Benayoun, B.A., Shi, Y., and Brunet, A. (2011). Transgenerational epigenetic inheritance of longevity in Caenorhabditis elegans. Nature *479*, 365–371.

Gregor, M.F., Misch, E.S., Yang, L., Hummasti, S., Inouye, K.E., Lee, A.-H., Bierie, B., and Hotamisligil, G.S. (2013). The role of adipocyte XBP1 in metabolic regulation during lactation. Cell Rep. *3*, 1430–1439.

Herquel, B., Ouararhni, K., Khetchoumian, K., Ignat, M., Teletin, M., Mark, M., Béchade, G., Van Dorsselaer, A., Sanglier-Cianférani, S., Hamiche, A., et al. (2011). Transcription cofactors TRIM24, TRIM28, and TRIM33 associate to form regulatory complexes that suppress murine hepatocellular carcinoma. Proc. Natl. Acad. Sci. USA *108*, 8212–8217.

Hollick, J.B., and Chandler, V.L. (2001). Genetic factors required to maintain repression of a paramutagenic maize pl1 allele. Genetics *157*, 369–378.

Jais, A., Einwallner, E., Sharif, O., Gossens, K., Lu, T.T.-H., Soyal, S.M., Medgyesi, D., Neureiter, D., Paier-Pourani, J., Dalgaard, K., et al. (2014). Heme oxygenase-1 drives metaflammation and insulin resistance in mouse and man. Cell *158*, 25–40.

Jakobsson, J., Cordero, M.I., Bisaz, R., Groner, A.C., Busskamp, V., Bensadoun, J.-C., Cammas, F., Losson, R., Mansuy, I.M., Sandi, C., and Trono, D. (2008). KAP1-mediated epigenetic repression in the forebrain modulates behavioral vulnerability to stress. Neuron *60*, 818–831.

Kamiya, M., Judson, H., Okazaki, Y., Kusakabe, M., Muramatsu, M., Takada, S., Takagi, N., Arima, T., Wake, N., Kamimura, K., et al. (2000). The cell cycle control gene ZAC/PLAGL1 is imprinted—a strong candidate gene for transient neonatal diabetes. Hum. Mol. Genet. *9*, 453–460.

Kiani, J., Grandjean, V., Liebers, R., Tuorto, F., Ghanbarian, H., Lyko, F., Cuzin, F., and Rassoulzadegan, M. (2013). RNA-mediated epigenetic heredity requires the cytosine methyltransferase Dnmt2. PLoS Genet. *9*, e1003498.

Koza, R.A., Nikonova, L., Hogan, J., Rim, J.-S., Mendoza, T., Faulk, C., Skaf, J., and Kozak, L.P. (2006). Changes in gene expression foreshadow diet-induced obesity in genetically identical mice. PLoS Genet. *2*, e81.

Körner, A., Kratzsch, J., Gausche, R., Schaab, M., Erbs, S., and Kiess, W. (2007). New predictors of the metabolic syndrome in children—role of adipocytokines. Pediatr. Res. *61*, 640–645.

Landgraf, K., Rockstroh, D., Wagner, I.V., Weise, S., Tauscher, R., Schwartze, J.T., Löffler, D., Bühligen, U., Wojan, M., Till, H., et al. (2015). Evidence of early alterations in adipose tissue biology and function and its association with obesity-related inflammation and insulin resistance in children. Diabetes *64*, 1249–1261.

Lee, H.-C., Gu, W., Shirayama, M., Youngman, E., Conte, D., Jr., and Mello, C.C.C. (2012). *C. elegans* piRNAs mediate the genome-wide surveillance of germline transcripts. Cell *150*, 78–87.

Messerschmidt, D.M., de Vries, W., Ito, M., Solter, D., Ferguson-Smith, A., and Knowles, B.B. (2012). Trim28 is required for epigenetic stability during mouse oocyte to embryo transition. Science *335*, 1499–1502.

Moon, Y.S., Smas, C.M., Lee, K., Villena, J.A., Kim, K.-H., Yun, E.J., and Sul, H.S. (2002). Mice lacking paternally expressed Pref-1/Dlk1 display growth retardation and accelerated adiposity. Mol. Cell. Biol. *22*, 5585–5592.

Morgan, H.D., Sutherland, H.G., Martin, D.I., and Whitelaw, E. (1999). Epigenetic inheritance at the agouti locus in the mouse. Nat. Genet. *23*, 314–318.

Morita, S., Horii, T., Kimura, M., Arai, Y., Kamei, Y., Ogawa, Y., and Hatada, I. (2014). Paternal allele influences high fat diet-induced obesity. PLoS ONE *9*, e85477.

Naumova, N., and Dekker, J. (2010). Integrating one-dimensional and three-dimensional maps of genomes. J. Cell Sci. *123*, 1979–1988.

O'Riain, M.J., Jarvis, J.U., and Faulkes, C.G. (1996). A dispersive morph in the naked mole-rat. Nature *380*, 619–621.

Öst, A., Lempradl, A., Casas, E., Weigert, M., Tiko, T., Deniz, M., Pantano, L., Boenisch, U., Itskov, P.M., Stoeckius, M., et al. (2014). Paternal diet defines offspring chromatin state and intergenerational obesity. Cell *159*, 1352–1364.

Ouchi, N., Parker, J.L., Lugus, J.J., and Walsh, K. (2011). Adipokines in inflammation and metabolic disease. Nat. Rev. Immunol. *11*, 85–97.

Patti, M.-E. (2013). Intergenerational programming of metabolic disease: evidence from human populations and experimental animal models. Cell. Mol. Life Sci. *70*, 1597–1608.

Pietiläinen, K.H., Naukkarinen, J., Rissanen, A., Saharinen, J., Ellonen, P., Keränen, H., Suomalainen, A., Götz, A., Suortti, T., Yki-Järvinen, H., et al. (2008). Global transcript profiles of fat in monozygotic twins discordant for BMI: pathways behind acquired obesity. PLoS Med. *5*, e51.

Pospisilik, J.A., Schramek, D., Schnidar, H., Cronin, S.J.F., Nehme, N.T., Zhang, X., Knauf, C., Cani, P.D., Aumayr, K., Todoric, J., et al. (2010). *Drosophila* genome-wide obesity screen reveals hedgehog as a determinant of brown versus white adipose cell fate. Cell *140*, 148–160.

Radford, E.J., Ito, M., Shi, H., Corish, J.A., Yamazawa, K., Isganaitis, E., Seisenberger, S., Hore, T.A., Reik, W., Erkek, S., et al. (2014). In utero effects. In utero undernourishment perturbs the adult sperm methylome and intergenerational metabolism. Science *345*, 1255903.

Rando, O.J., and Simmons, R.A. (2015). I'm eating for two: parental dietary effects on offspring metabolism. Cell *161*, 93–105.

Rassoulzadegan, M., Grandjean, V., Gounon, P., Vincent, S., Gillot, I., and Cuzin, F. (2006). RNA-mediated non-mendelian inheritance of an epigenetic change in the mouse. Nature *441*, 469–474.

Rechavi, O., Houri-Ze'evi, L., Anava, S., Goh, W.S.S., Kerk, S.Y., Hannon, G.J., and Hobert, O. (2014). Starvation-induced transgenerational inheritance of small RNAs in *C. elegans*. Cell *158*, 277–287.

Rowe, H.M., Jakobsson, J., Mesnard, D., Rougemont, J., Reynard, S., Aktas, T., Maillard, P.V., Layard-Liesching, H., Verp, S., Marquis, J., et al. (2010). KAP1 controls endogenous retroviruses in embryonic stem cells. Nature *463*, 237–240.

Schotta, G., Ebert, A., Dorn, R., and Reuter, G. (2003). Position-effect variegation and the genetic dissection of chromatin regulation in Drosophila. Semin. Cell Dev. Biol. *14*, 67–75.

Sekita, Y., Wagatsuma, H., Nakamura, K., Ono, R., Kagami, M., Wakisaka, N., Hino, T., Suzuki-Migishima, R., Kohda, T., Ogura, A., et al. (2008). Role of retrotransposon-derived imprinted gene, Rtl1, in the feto-maternal interface of mouse placenta. Nat. Genet. *40*, 243–248.

Seong, K.-H., Li, D., Shimizu, H., Nakamura, R., and Ishii, S. (2011). Inheritance of stress-induced, ATF-2-dependent epigenetic change. Cell *145*, 1049–1061.

Shirayama, M., Seth, M., Lee, H.-C., Gu, W., Ishidate, T., Conte, D., Jr., and Mello, C.C. (2012). piRNAs initiate an epigenetic memory of nonself RNA in the *C. elegans* germline. Cell *150*, 65–77.

Simpson, S.J., Sword, G.A., and Lo, N. (2011). Polyphenism in insects. Curr. Biol. *21*, R738–R749.

Smith, F.M., Holt, L.J., Garfield, A.S., Charalambous, M., Koumanov, F., Perry, M., Bazzani, R., Sheardown, S.A., Hegarty, B.D., Lyons, R.J., et al. (2007). Mice with a disruption of the imprinted Grb10 gene exhibit altered body composition, glucose homeostasis, and insulin signaling during postnatal life. Mol. Cell. Biol. *27*, 5871–5886.

Teperino, R., Amann, S., Bayer, M., McGee, S.L., Loipetzberger, A., Connor, T., Jaeger, C., Kammerer, B., Winter, L., Wiche, G., et al. (2012). Hedgehog partial agonism drives Warburg-like metabolism in muscle and brown fat. Cell *151*, 414–426.

Varrault, A., Gueydan, C., Delalbre, A., Bellmann, A., Houssami, S., Aknin, C., Severac, D., Chotard, L., Kahli, M., Le Digarcher, A., et al. (2006). Zac1 regulates an imprinted gene network critically involved in the control of embryonic growth. Dev. Cell *11*, 711–722.

Vrang, N., Meyre, D., Froguel, P., Jelsing, J., Tang-Christensen, M., Vatin, V., Mikkelsen, J.D., Thirstrup, K., Larsen, L.K., Cullberg, K.B., et al. (2010). The imprinted gene neuronatin is regulated by metabolic status and associated with obesity. Obesity (Silver Spring) *18*, 1289–1296.

Waterland, R.A., Dolinoy, D.C., Lin, J.-R., Smith, C.A., Shi, X., and Tahiliani, K.G. (2006). Maternal methyl supplements increase offspring DNA methylation at Axin Fused. Genesis *44*, 401–406.

Whitelaw, N.C., Chong, S., Morgan, D.K., Nestor, C., Bruxner, T.J., Ashe, A., Lambley, E., Meehan, R., and Whitelaw, E. (2010). Reduced levels of two modifiers of epigenetic gene silencing, Dnmt3a and Trim28, cause increased phenotypic noise. Genome Biol. *11*, R111.

WHO (2015). Obesity Fact Sheet N°311. http://www.who.int/mediacentre/factsheets/fs311/en/.

Wolff, G.L., Kodell, R.L., Moore, S.R., and Cooney, C.A. (1998). Maternal epigenetics and methyl supplements affect agouti gene expression in Avy/a mice. FASEB J. *12*, 949–957.

Yang, J., Loos, R.J.F., Powell, J.E., Medland, S.E., Speliotes, E.K., Chasman, D.I., Rose, L.M., Thorleifsson, G., Steinthorsdottir, V., Mägi, R., et al. (2012). FTO genotype is associated with phenotypic variability of body mass index. Nature *490*, 267–272.

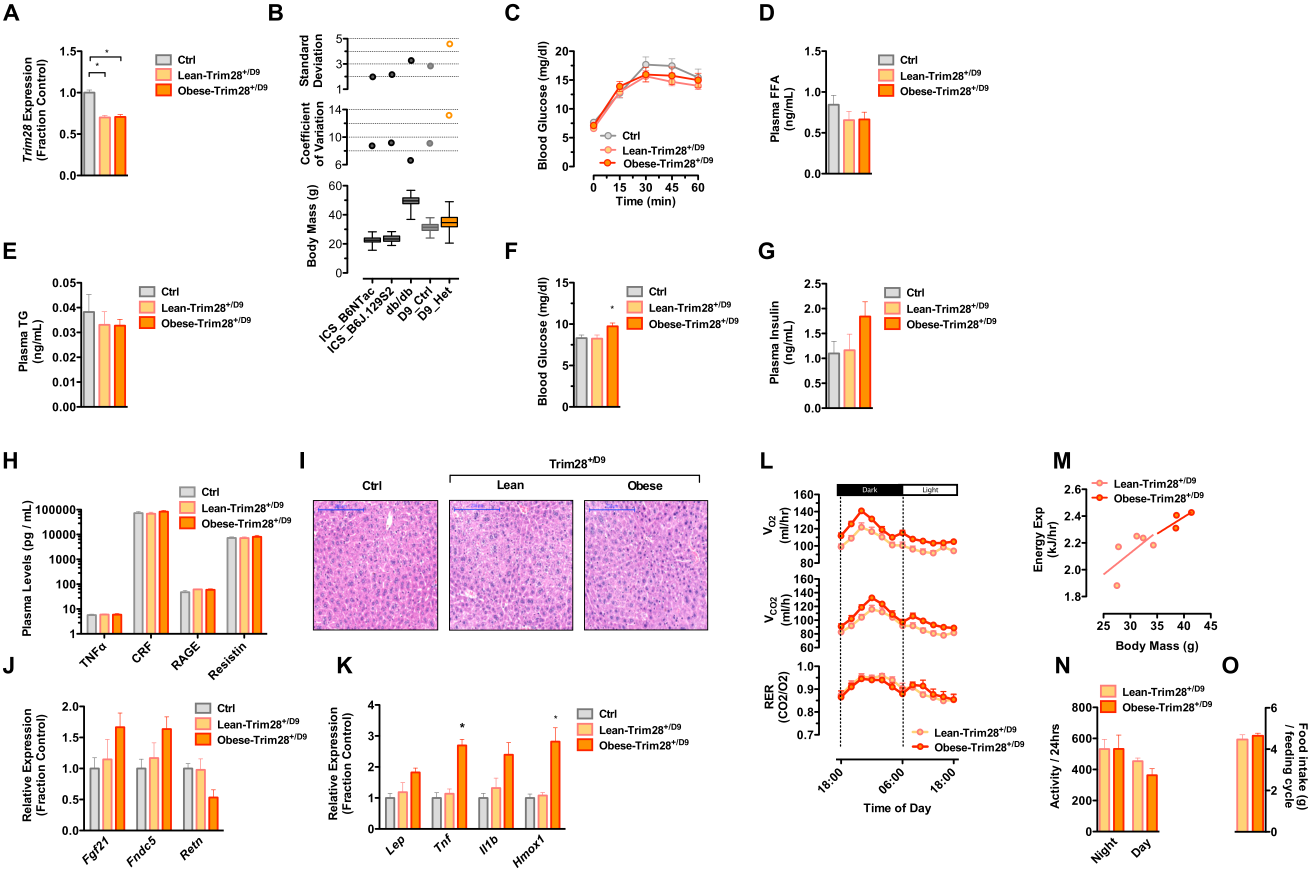

Figure S1. Obese-Trim28$^{+/D9}$ Animals Are Metabolically “Healthy,” Related to Figure 1

(A) *Trim28* is reduced and equally expressed in lean- and obese-Trim28$^{+/D9}$ animals relative to wild-type littermates.

(B) Comparison of body mass, coefficient of variation, and standard deviation against large, independently obtained, non-early-life-controlled cohorts confirmed the heightened variation specifically of the Trim28$^{+/D9}$ heterozygotes.

(C–H) Assessment of metabolic health by OGTT (C), shows that both lean- and obese-Trim28$^{+/D9}$ exhibit unaltered glucose homeostasis and plasma levels of (D) FFA and (E) triglycerides. Obese-Trim28$^{+/D9}$ animals showed (F) slightly higher blood glucose levels after 6 hr fast and (G) elevated serum levels of insulin. (H) Plasma levels of adipokines known to be associated with metabolically diseased obesity were unremarkable.

(I) H&E staining of liver sections (scale bar: 200μm) revealed no sign of hepatosteatosis in *Trim28* haploinsufficient animals consistent with being metabolic healthy.

(J and K) Trim28$^{+/D9}$-induced obesity was associated with moderate expression changes with both (J) positive and (K) negative metabolic outcomes.

(L–O) Indirect calorimetry in obese- and lean-Trim28$^{+/D9}$ animals show changes in (L) V_{O2}, V_{CO2} and (M) energy expenditure (n = 3–5). The changes are associated with trend toward reduced (N) activity and (O) unaltered food intake.

Data are mean ± SEM (*$p < 0.05$).

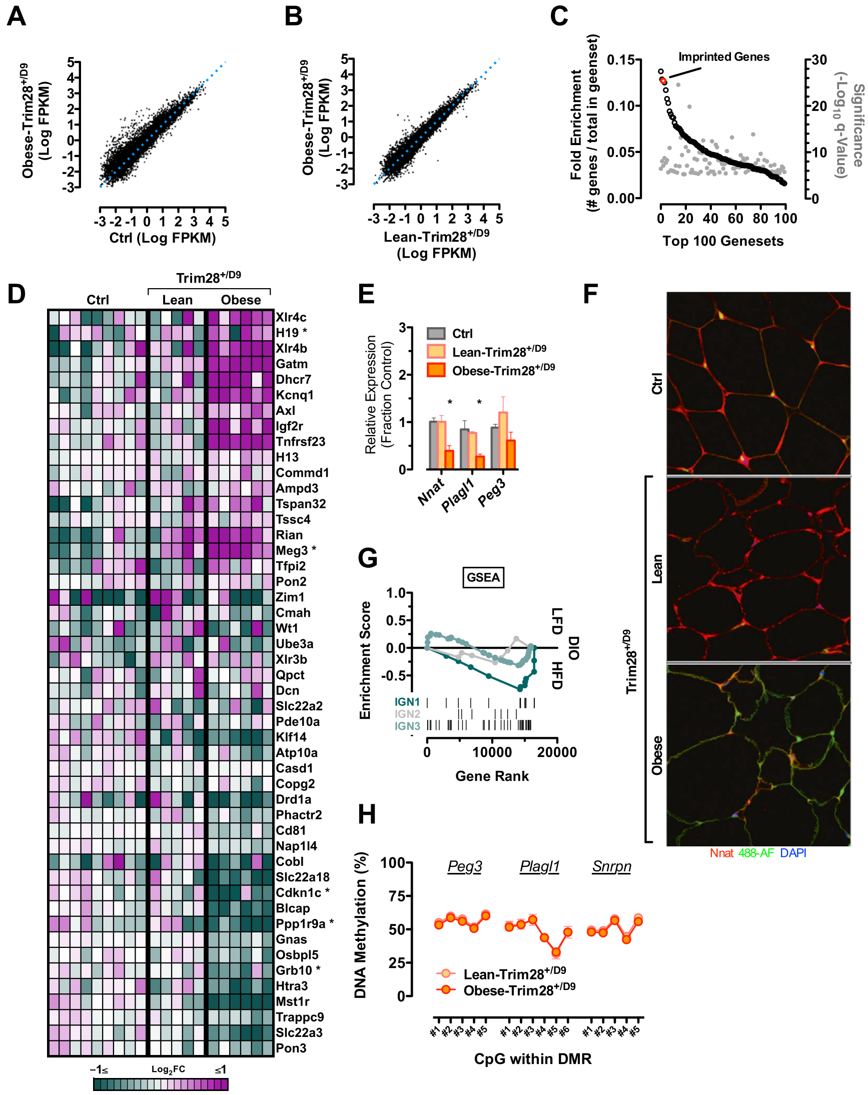

Figure S2. Non-classical Imprinted Gene Dysregulation in Trim28$^{+/D9}$-Induced Obesity, Related to Figure 2

(A and B) Correlation of RNA-seq data of epididymal adipose tissue from obese-Trim28$^{+/D9}$ animals with either (A) wild-type or (B) lean-Trim28$^{+/D9}$ littermates.

(C) "Imprinted genes" is the 3rd most discordant gene set when comparing high-fat diet induced and Trim28$^{+/D9}$-sensitized obesity.

(D) Heatmap of gene expression changes to maternally expressed imprinted genes.

(E) Reduced expression of *Nnat*, *Plagl1*, and *Peg3* in obese-Trim28$^{+/D9}$ animals was confirmed by qPCR.

(F) Nnat was also found reduced at the protein level when assessed by immunofluorescence. DAPI is shown in blue, Nnat in red and 488-AF (autofluorescence) in green.

(G) GSEA of diet-induced obesity adipose tissue mRNA expression data reveal, if anything, an opposite regulation of IGN1.

(H) Bisulfite pyrosequencing of germline DMR's in stromal vascular adipocyte progenitor preparations from lean- and obese-Trim28$^{+/D9}$ showed no differences. Data are mean ± SEM (*$p < 0.05$).

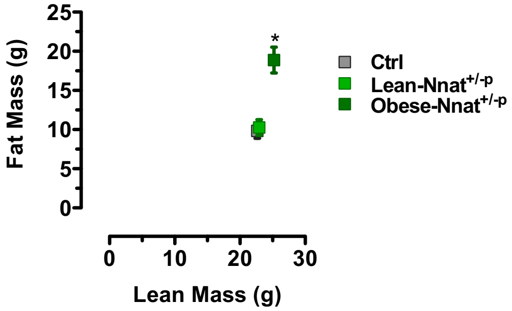

Figure S3. Increased Adiposity in Obese-Nnat$^{+/-p}$ Mice, Related to Figure 3

Dual-emission X-ray absorption analysis for lean and fat mass in heavy and light paternal *Nnat* deletion mutants. Means of each group are shown as squares. Data are mean ± SEM (*$p < 0.05$).

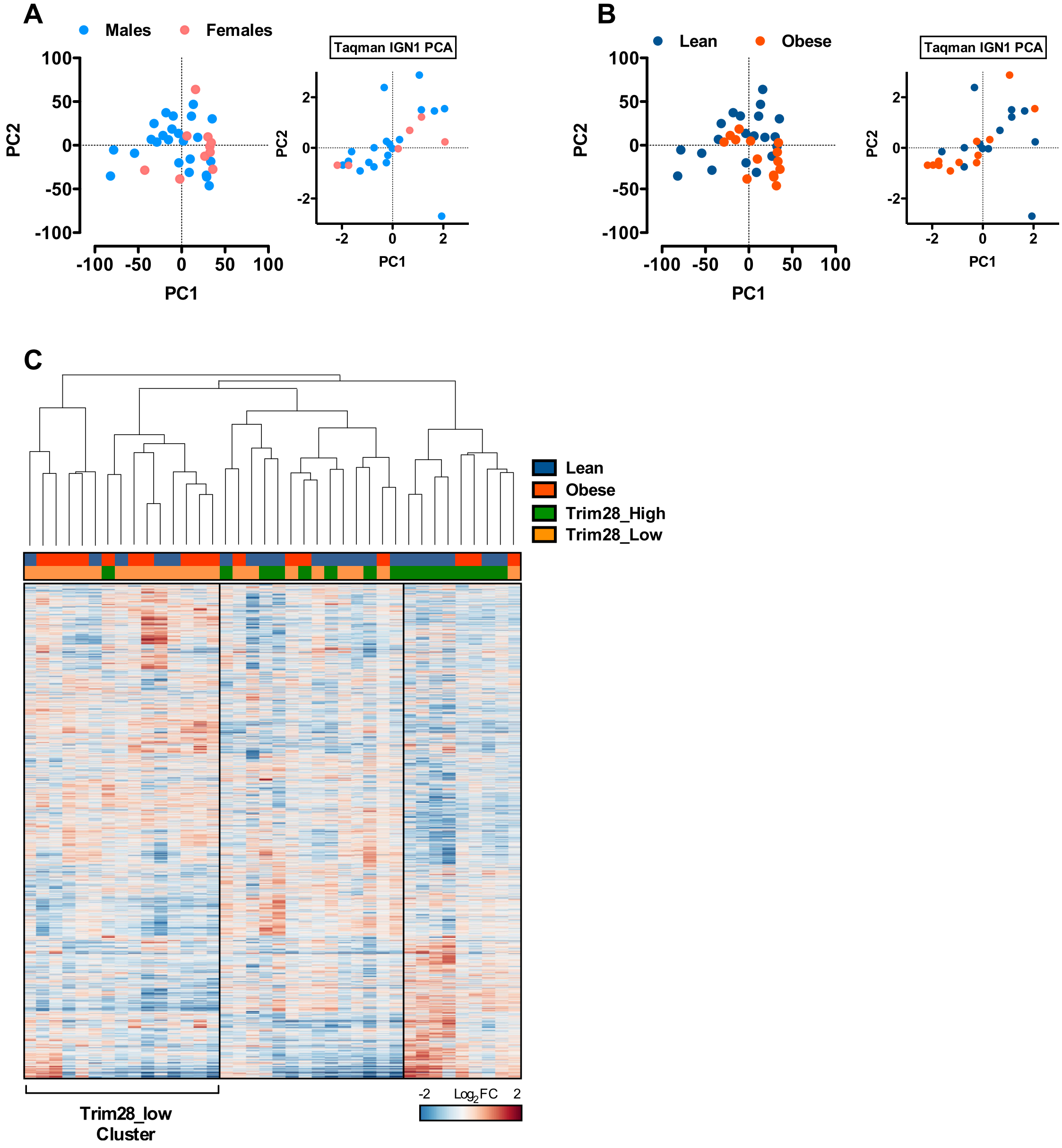

Figure S4. PCA of Human Childhood Adipose Tissue Transcriptome Data, Related to Figure 4

(A and B) PCA of adipose tissue RNA-seq from children of the Leipzig AT cohort revealed Tim28-low versus -high individuals to be substantially different. Visualization of (A) males versus females, or (B) obese versus lean individuals does not yield the same group segregation. Insets highlight the same to be true when analyzing on IGN1 qPCR expression data.

(C) Hierarchical clustering of the heatmap visualization of the 6,000 most variable, expressed genes reveals the relative genic sub-structure of the major clusters including the major Trim28-low and -high stratified clusters. Vertical lines are for visualization purposes only.

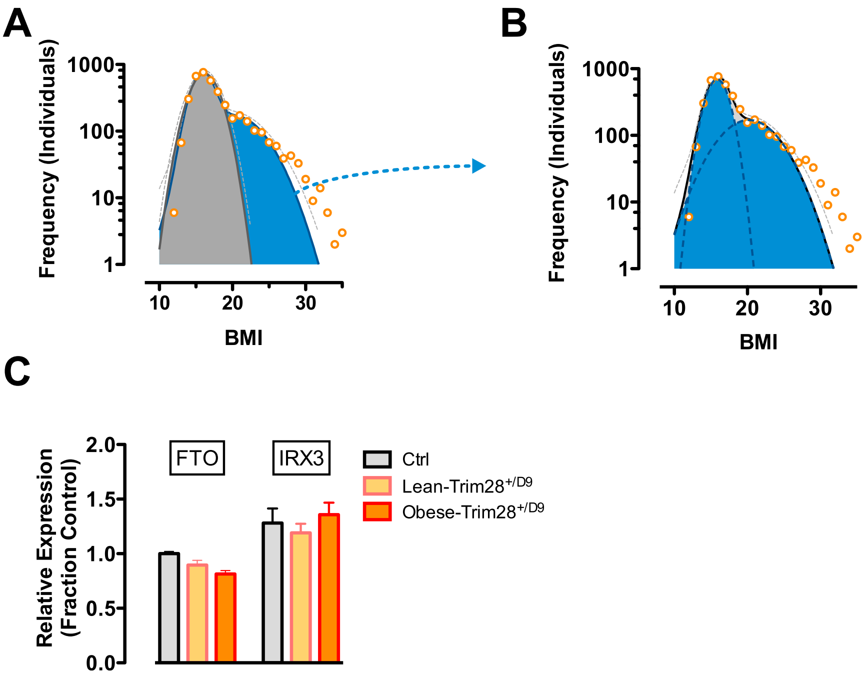

Figure S5. NHANES Population BMI Distributions, Related to Figure 5

(A) BMI distribution of 6- to 11-year-old non-hispanic white male participants from the continuous NHANES 1999–2012 survey (CDC, 2012). Data are fit to a single Gaussian (gray) and a double Gaussian (blue) and visualized on a log-scale to highlight data from the more rare, morbidly obese individuals that avoid the fit.

(B) Individual Gaussian components of the double Gaussian from (A).

(C) *FTO* expression is significantly reduced in obese-Trim28$^{+/D9}$ animals.

Data are mean ± SEM.

Cell

Supplemental Information

Trim28 Haploinsufficiency Triggers Bi-stable Epigenetic Obesity

Kevin Dalgaard, Kathrin Landgraf, Steffen Heyne, Adelheid Lempradl, John Longinotto, Klaus Gossens, Marius Ruf, Michael Orthofer, Ruslan Strogantsev, Madhan Selvaraj, Tess Tsai-Hsiu Lu, Eduard Casas, Raffaele Teperino, M. Azim Surani, Ilona Zvetkova, Debra Rimmington, YC Loraine Tung, Brian Lam, Rachel Larder, Giles SH Yeo, Stephen O'Rahilly, Tanya Vavouri, Emma Whitelaw, Josef M. Penninger, Thomas Jenuwein, Ching-Lung Cheung, Anne C. Ferguson-Smith, Anthony P. Coll, Antje Körner, and J. Andrew Pospisilik

SUPPLEMENTAL EXPERIMENTAL PROCEDURES

Animal Husbandry

All mice were maintained under controlled temperature (22°C) and on a 12hr light, 12hr dark schedule (light on 6:00-18:00). Food and water were available *ad libitum* unless otherwise stated. All mice were weaned at 3 weeks of age onto a standard chow (SDS RM3, Essex, UK or V1185-300 MZ-Ereich, ssniff, Germany). Body composition was determined using dual-energy X-ray absorptiometry (DEXA, PIX-Imus2 Series Densimeters) under terminal anaesthetic (Dolethal, Vetoquinol UK Ltd). All protocols were in accordance with German and United Kingdom legislation; Project license numbers 80/2098, 80/2497, and 35-9185.81/G-10/94.

Generation of *Trim28* and *Nnat* Heterozygous Mice

The generation of Trim28$^{+/D9}$ is described elsewhere (Blewitt et al, 2005 and Whitelaw et al, 2010). Briefly, the line was generated by ENU mutation in an FVB/NJ congenic line expressing a GFP transgene array (Whitelaw et al 2010). This mouse line was outcrossed 2 times to C57BL6/J for mutation mapping, then, backcrossed to FVB/NJ for more than 10 generations and inbred another 20+ generations. The Nnat targeting vector was designed to produce a "KO first allele" (reporter-tagged insertion with conditional potential) (Figure 3A). A Gateway adapted intermediate vector containing Nnat homology arms and the critical exons 2 and 3 was obtained from The Wellcome Trust Sanger Institute high throughput gene targeting pipeline, design ID 44045 (http://www.sanger.ac.uk/htgt/htgt2/design/designlist/list _designs). A Gateway reaction was assembled with Nnat-interm_44045 vector, pL3L4_DONR223_Spec_DTA(-) Terminator and pL1L2_GTIRES _BetactP_FLFL provided by Dr B. Rosen (The Wellcome Trust Sanger Institute). The resulting vector was used to target JM8.F6 C57Bl6/N embryonic stem cells cultured according to standard protocols as recommended by the International Knockout Mouse Consortium. Confirmation of targeting was by PCR with primer sets 21_16 and 9_19 (available on request). Targeting efficiency was 40%.

Proper targeting was confirmed for clones A6 and B12 by Southern blot and clones were injected into to C57BL/6J-Tyr^{c-2J} (albino) blastocysts. Chimeric mice were then mated with C57BL/6J-Tyr^{c-2J} mice and germ line transmission was validated by the appearance of black offspring. The founder mice were then crossed with C57BL/6J mice. Targeted mice were genotyped by PCR using primer sets 55_56_57 and 59_60_61 (available on request). Mice heterozygous for the "KO first allele" were crossed with CMVCre C57BL/6J mice (kind gift from Dr M. Constancia) to delete the *neo* cassette and exons 2 and 3 from the targeted *Nnat* genomic locus. Primer set 82_83 was used to

confirm recombination between loxP site 1 and loxP site 3. Mice carrying the *Nnat* KO allele were crossed with C57BL/6J mice to breed the *Cre* transgene out. qPCR and Western blotting were used to confirm the lack of *Nnat* expression in the targeted mice (Figures 3B and 3C). $Nnat^{+/-}$ mice were backcrossed for 10 generations onto a C57BL/6J background before undergoing phenotypic analysis. $Nnat^{+/-p}$ animals were generated by crossing wild-type females with male $Nnat^{+/-}$ mice. $Nnat^{+/-m}$ mice were generated by crossing $Nnat^{+/-}$ females with wild-type male mice. $Nnat^{+/-m}$ mice were phenotypically identical to $Nnat^{+/+}$ mice (Figure 3D).

Generation of *Trim28* Tissue-specific Knockout Mice

The generation of animals harboring conditional *Trim28* alleles has been previously described (Cammas et al, 2000). Constitutive gene inactivation in tissues was performed using transgenic Alb-Cre (liver), McK-Cre (muscle), adipose (Adipoq-Cre), POMC (POMC-Cre) and AgRP (AgRP-Cre) animals obtained from The Jackson Laboratory. To generate tissue-specific knockouts, $Trim28^{flox/flox}$ animals were crossed with Cre-positive $Trim28^{+/flox}$ animals to obtain mice homozygous for the floxed *Trim28* locus. These crosses produce Cre-positive-control and tissue-specific heterozygote and knockout littermates. Data shown are from 14-18 weeks old animals.

Quantitative PCR

Total RNA was extracted using TRI Reagent (SIGMA) and was reverse-transcribed into cDNA using commercially available kits (Applied Biosystems). qPCR reactions were performed a 7900HT Fast Real-Time PCR System (Applied Biosystems). Post-amplification melting curve analysis was performed to check for unspecific products and primer-only controls were included to ensure the absence of primer dimers. For normalization threshold cycles (Ct-values) were normalized to either TBP (mouse samples) or to ACTB, HPRT and TBP (human samples) within each sample to obtain sample-specific ΔCt values (= Ct *gene of interest* - Ct *housekeeping gene*). $2^{-\Delta\Delta Ct}$ values were calculated to obtain fold expression levels, where ΔΔCt = (ΔCt treatment - ΔCt control). Human transcripts were quantified using TaqMan gene expression assays with validated probes (Life Technologies). Human and mouse primers used are listed in Supplemental Table S5.

Glucose Tolerance Test

Following an overnight fast, mice were administered glucose (1 g/kg) by oral gavage, and blood samples for glucose measurement were collected from the tail vein at the indicated times. Glycemia was assessed using a OneTouch Vita glucometer.

Mouse Laboratory Parameters and Cytokines

Free fatty acids and glycerol were measured using the non-esterified fatty acid (NEFA) kit (Wako Chemicals) and Triglyceride Determination kit (SIGMA), respectively, and the provided protocol was followed. Serum leptin, TNFα, CRF, RAGE and Resistin levels were quantified using commercially available ELISA kits (Millipore, R&D Systems).

Histology, Adipocyte Size and Number

For tissue sections, hematoxylin and eosin (H&E) staining was performed on 2 µm paraffin sections of tissues fixed in 4% phosphate-buffered formalin. Adipocyte size distribution was determined by semi-automated morphometry. In brief, whole fat pads were visualized using whole slide scans captured by the Mirax Scan Pannoramic Scanner with a 20x objective (Zeiss and 3DHistec). Four fields of view of 200 µm intervals per animal were quantified. Epididymal fat pads from 4 animals per group were analyzed. Semi-automated morphometry (ImageJ, plugin Adipocytes Tool) was used to define and quantify adipocytes based on shape, size and presence of a lipid droplet.

Immunofluorescence

Paraffinized sections were heated, deparaffinized and rinsed in water. Antigen retrieval was performed by heating the slides at 95°C for 20 minutes in HistoVT pH 7.0 (Nacalai USA). Specimens were blocked in 5% goat serum PBS-T and incubated with Nnat primary antibody (1:50 dilution) (AB27266, Abcam) for 48 hours. Specimens were incubated in fluorochrome-conjugated secondary antibody. VectorShield with DAPI and coverslip were mounted and slides were allowed to cure overnight at 4°C in the dark before image acquisition. Images were acquired using LSM780 (Zeiss).

RNA Sequencing

Trizol-purified RNA was poly(A)-enriched and libraries prepared using the TruSeq Sample Prep v2 kit (Illumina) and sequenced on an Illumina HiSeq 2500 sequencer. All mouse sequence data were performed in at least three biological replicates at 2 x 50bp length with high quality metrics (> 20 Phred score) and nucleotide distribution. > 15 million reads per mouse sample and 10 million reads per human sample were mapped using TopHat v2.0.8b with -G option against *Mus musculus* genome (mm9, iGenome UCSC) and *Homo sapiens* genome (hg19, iGenome UCSC), respectively. Gene expression values and significantly differentially expressed genes were calculated using Cuffnorm and Cuffdiff v2.2.1 (Trapnell et al., 2013) with geometric normalization and multi-read correction (-u option).

Reduced Representative Bisulfite Sequencing

Total genomic DNA was digested over night with MspI (NEB) and sequencing libraries were prepared with NEBNext DNA Library kit (NEB). Libraries were size-selected (150-350 bp) on an agarose gel followed by bisulfite conversion using Imprint DNA Modification kit (SIGMA) using the 2-step protocol (2 hr at 65°C). Samples were PCR amplified using KAPA Hifi Uracil+ high-fidelity polymerase (KAPA Biosystems). Library quality was assessed using KAPA Library Quantification kit (KAPA BIosystem) and sequenced on an Illumina HiSeq 2500 sequencer. Data were analyzed using RnBeads V.0.99.16 (Assenov et al., 2014).

Quantitative Bisulfite Pyrosequencing

Total genomic DNA was bisulfite converted using Imprint DNA Modification kit (SIGMA) using the 2-step protocol (2 hr at 65°C). Samples were PCR amplified for the region of interest and bound to streptavidin beads using PyroMark Binding Buffer (Qiagen). Beads were washed in 70% Ethanol, 0.4N NaOH and 10mM Tris-acetate (pH 7.6) using a PyroMark Vacuum workstation (Qiagen). DNA-bound beads were resuspended in PyroMark annealing buffer (Qiagen) containing 10uM Pyrosequencing primer (PSQ, Qiagen). Samples were heated up and sequenced on a PyroMark Q96 PyroSequencer (Qiagen) in triplicates and were analysed using PyroMark Q96 ID Software 2.5.

Human Study Population

Study subjects of the Leipzig Childhood adipose cohort (NCT02208141) included 18 obese patients and 22 lean controls aged 2-18 years that underwent elective orthopedic surgery, herniotomy/orchidopexie or other surgeries (Landgraf et al., 2015). Participants were included if they were free of severe diseases and medication potentially affecting adipose tissue biology. The following exclusion criteria were applied: diabetes, generalized inflammation, malignant diseases, genetic syndromes, or permament immobilization. Written informed consent was obtained from all parents and study protocols were approved by the local Ethics Committee (265–08, 265–08-ff). BMI data of study participants were standardized to age- and sex-specific centiles applying German reference data (Kromeyer-Hauschild et al., 2001). Overweight and obesity are defined by a cutoff of 1.28 and 1.88 SDS (90th or 97th centile), respectively. Three overweight and 15 obese subjects were combined to form the “Obese” group. Tissue biopsies from subcutaneous adipose tissue were excised during surgery, washed three times in PBS, and immediately frozen in liquid nitrogen for RNA isolation.

Reagents

Unless otherwise stated all chemicals and reagents were obtained from SIGMA.

Statistical Analysis

Data are expressed as mean ± standard error of the mean (SEM) unless otherwise specified. Statistical significance was tested by Student's t-test or ANOVA where appropriate. Correlations were tested by linear regression. All figures and mouse statistical analyses were generated using Prism 5 (GraphPad). All reported p-values are two-tailed unless stated otherwise.
$p < 0.05$ was considered to indicate statistical significance.

Supplemental References

Assenov, Y., Müller, F., Lutsik, P., Walter, J., Lengauer, T., and Bock, C. Comprehensive analysis of DNA methylation data with RnBeads. *Nat Methods* 11, 1138-140 (2014).

Cammas F, Mark M, Dollé P, Dierich A, Chambon P, Losson R. Mice lacking the transcriptional corepressor TIF1beta are defective in early postimplantation development. *Development. 2000 Jul;127(13):2955-63.*

Kromeyer-Hauschild, K., Wabitsch, M., Kunze, D., Geller, F., Geiß, H.C., Hesse, V., von Hippel, A., Jaeger, U., Johnsen, D., and Korte, W. Perzentile für den Body-mass-Index für das Kindes-und Jugendalter unter Heranziehung verschiedener deutscher Stichproben. *Monatsschrift Kinderheilkunde* 149, 807-818 (2001).

Landgraf, K., Rockstroh, D., Wagner, I.V., Weise, S., Tauscher, R., Schwartze, J.T., Löffler, D., Bühligen, U., Wojan, M., Till, H., Kratzsch, J., Kiess, W., Blüher, M., and Körner, A. Evidence of early alterations in adipose tissue biology and function and its association with obesity-related inflammation and insulin resistance in children. *Diabetes* 64, 1249-261 (2015).

Trapnell, C., Hendrickson, D.G., Sauvageau, M., Goff, L., Rinn, J.L., and Pachter, L. Differential analysis of gene regulation at transcript resolution with RNA-seq. *Nat Biotechnol* 31, 46-53 (2013).
